# Oxidative stress promotes liver fibrosis by modulating the microRNA-144 and SIN3A-p38 pathways in hepatic stellate cells

**DOI:** 10.7150/ijbs.92749

**Published:** 2024-04-08

**Authors:** Yawen Hao, Shaohua Song, Tao Li, Qiuhong Zai, Ningning Ma, Yixin Li, Liu Yang, Peng Xiao, Tianyue Xu, Longshan Ji, Jiaxin Tan, Yeni Ait Ahmed, Xiaogang Xiang, Xiaolin Wang, Fouad Lafdil, Qing Xie, Yong He

**Affiliations:** 1 Shanghai Institute of Materia Medica (SIMM), Chinese Academy of Sciences, Shanghai, China.; 2 University of Chinese Academy of Sciences, Beijing, China.; 3 Department of General Surgery or Department of Infectious Diseases, Ruijin Hospital, Shanghai Jiao Tong University School of Medicine, Shanghai, China.; 4 Department of Cardiology, The First Affiliated Hospital of USTC, Division of Life Sciences and Medicine, University of Science and Technology of China (USTC), Hefei, China.; 5 Department of Hepatology, First Hospital of Jilin University, Jilin University, Changchun, Jilin, China.; 6 Laboratory of Cellular Immunity, Shanghai Key Laboratory of Traditional Chinese Medicine, Shuguang Hospital Affiliated to Shanghai University of Traditional Chinese Medicine, Shanghai, China.; 7 Medizinische Klinik m. S. Hepatologie und Gastroenterologie Charité Universitätsmedizin Berlin, Germany.; 8 Université Paris-Est-Créteil (UPEC), F-94000, Créteil, France.; 9 Institut Universitaire de France (IUF), Paris, F-75231 Cedex 05 France.

**Keywords:** AAV6, ROS, HSC, p38, liver fibrogenesis

## Abstract

**Background & Aims**: Reactive oxygen species (ROS) act as modulators triggering cellular dysfunctions and organ damage including liver fibrosis in which hepatic stellate cell (HSC) activation plays a key role. Previous studies suggest that microRNA-144 (miR-144) acts as a pro-oxidant molecule; however, whether and how miR-144 affects HSC activation and liver fibrosis remain unknown.

**Methods:** Carbon tetrachloride (CCl_4_) and bile duct ligation (BDL)-induced experimental liver fibrosis models were used. Hepatic miR-144 expression was analyzed by miRNA *in situ* hybridization with RNAscope probe. The *in vivo* effects of silencing or overexpressing miR-144 were examined with an adeno-associated virus 6 (AAV6) carrying miR-144 inhibitor or mimics in fibrotic mouse experimental models.

**Results:** In this study, we demonstrated that ROS treatment significantly upregulated miR-144 in HSCs, which further promoted HSC activation *in vitro*. Interestingly, miR-144 was preferentially elevated in HSCs of experimental liver fibrosis in mice and in human liver fibrotic tissues. Furthermore, *in vivo* loss or gain-of-function experiments via AAV6 carrying miR-144 antagomir or agomir revealed that blockade of miR-144 in HSCs mitigated, while overexpression of miR-144 in HSCs accelerated the development of experimental liver fibrosis. Mechanistically, SIN3 transcription regulator family member A (SIN3A), a transcriptional repressor, was identified to be the target of miR-144 in HSCs. MiR-144 downregulated *Sin3A*, and in line with this result, specific knockdown of *Sin3a* in HSCs remarkedly activated p38 MAPK signaling pathway to promote HSC activation, eventually exacerbating liver fibrosis.

**Conclusions:** Oxidative stress-driven miR-144 fuels HSC activation and liver fibrogenesis by limiting the SIN3A-p38 axis. Thus, a specific inhibition of miR-144 in HSCs could be a novel therapeutic strategy for the treatment of liver fibrosis.

## Introduction

Liver fibrosis is a pathophysiological process of dysregulated liver repair caused by persistent liver injury, manifested by the deposition of the extracellular matrix (ECM) and generation of the fibrous scar 1. A variety of liver diseases, such as hepatitis B/C, alcoholic hepatitis, nonalcoholic steatohepatitis (NASH), and biliary tract disease, are responsible for the development of liver fibrosis 2. Importantly, patients undergoing fibrogenic progression may further develop cirrhosis and hepatocellular carcinoma (HCC), contributing to a significant global health burden 3. However, liver fibrosis is recognized to be regressive, as observed clinically in patients with hepatitis B infection or alcohol-related liver disease 4, 5. Therefore, elucidating the detailed mechanism of liver fibrosis will facilitate the exploitation of anti-fibrotic strategies.

Activated hepatic stellate cells (HSCs) are well believed to be the key effectors of liver fibrogenesis by producing matrix proteins during chronic liver injury. Therefore, there is an exigent need to clarify the cellular and molecular mechanisms of HSC activation in order for therapeutics to mimic the liver's endogenous capacity. In contrast to the quiescent phenotype in normal liver, HSCs transdifferentiate into myofibroblasts after liver injury, which is characterized by ECM accumulation 1, 6. HSC activation is stimulated by damaged and apoptotic hepatocytes through several converging pathways, which initiate and maintain the activation of HSCs by recruitment of immune cells, release of reactive oxygen species (ROS) and other fibrogenic/proinflammatory mediators, and disruption of the normal ECM of the space of Disse 5, 7. After ~40 years of steady progress in basic, translational and clinical research, there is a rich appreciation of the mechanism of HSC activation and the pathogenesis of liver fibrosis. Yet, achievement in treating liver fibrosis has been harder won than anyone anticipated since there is still no FDA-approved drugs for the treatment of liver fibrosis. Eventually, attention has focused on the profibrotic microenvironment of the liver and cell-cell interactions. Notably, oxidative stress in fibrotic microenvironment is identified to be a significant feature of HSC activation in chronic liver diseases 8. For example, Kupffer cells, liver-specific resident macrophages, are proven to be the main effectors responsible for the generation of ROS, contributing to HSC activation when exposed to harmful stimuli 9. Additionally, liver infiltrated neutrophils promote HSC activation by releasing ROS through an “oxidative burst” 10, 11. Although emerging evidence indicate that oxidative stress in fibrotic microenvironment affects HSC activation, the exact mechanism remains obscure.

MicroRNAs (miRNAs) is an endogenous, non-coding small RNA, 18-24 nucleotides in length, which regulate gene expression at the post-transcriptional levels by binding to the 3' untranslated region (UTR) to modulate abundant biological processes 12. Recent studies have revealed that ROS affect the expression levels of various miRNAs by modulating their biogenesis, translocation, and maturation 13. More importantly, ROS-driven miRNAs affect disease progression 13. Among these dysregulated miRNAs, miR-144 seems to be more sensitive to oxidative stress stimulation. For instance, oxidative stress can drive the production of miR-144 and further weaken the anti-oxidative stress response 14, 15. However, whether oxidative stress drives miR-144 production in HSCs, thus affecting HSC activation and liver fibrogenesis remains unknown.

In the current study, we demonstrated that oxidative stress induction resulted in miR-144 elevation, which was closely related to HSC activation. Most interestingly, miR-144 was preferentially observed in HSCs *in vivo* during liver fibrosis progression. Gain or loss-of-function data *in vivo* suggest that specific blockade of miR-144 in HSCs mitigated, overexpression of miR-144 in HSCs accelerated the development of experimental liver fibrosis by limiting the SIN3A-p38 axis. Taken together, oxidative stress-driven miR-144 is a crucial regulator to fuel HSC activation and specifically targeting miR-144 may provide a novel therapeutic strategy for the treatment of liver fibrosis.

## Materials and Methods

### Mice and experimental liver fibrosis models

8-week male C57BL/6J mice were purchased from the Jackson Laboratory (Bar Harbor, ME) or Beijing Vital River Laboratory Animal Technology Co., Ltd. For the carbon tetrachloride (CCl_4_)-induced liver fibrosis, mice were injected intraperitoneally with 2 ml/kg weight of 10% CCl_4_ (MACKLIN, catalog O815211, Shanghai, China) in olive oil (MACKLIN, catalog C805325, Shanghai, China) twice a week for 6 weeks. For the Adeno-Associated Virus 6 (AAV6)-miR-144 agomir/antagomir-treated experiments, mice were pretreated with CCl_4_ for 2 weeks before administering AAV6-miR-144 agomir (2.5×10^11^ viral genomes; OBiO, Shanghai, China) /antagomir (3.0×10^11^ viral genomes; OBiO, Shanghai, China) or AAV6-scramble (non-targeting control) (2.5×10^11^ viral genomes; OBiO, Shanghai, China) via tail vein injection. After AAV6 injection, another 8 doses of CCl_4_ were administered to the mice, which were sacrificed 48 hours after the last injection of CCl_4_. For bile duct ligation (BDL)-induced liver fibrosis, BDL or sham operation was performed as previously documented 16. Briefly, the incision was made along the midline of the abdomen while the mouse was anesthetized. The common bile duct was separated and double-ligated with surgical thread (4-0 silk). Mice in the sham-operated group underwent the same procedure except for bile duct ligation. AAV6-scramble, AAV6-miR-144 antagomir, and AAV6-miR-144 agomir were administered to mice via the tail vein three days after surgery. The duration of the experiment lasted 17 days after surgery. All experiments were performed according to institutional animal care ethics guidelines and approved by the Institutional Animal Care and Use Committee (IACUC) of Shanghai Institute of Materia Medica, Chinese Academy of Sciences.

### Human samples

Liver cirrhosis patients (n=5) who were subjected to liver transplantation, and underwent liver biopsy for the diagnoses were obtained from the Ruijin Hospital, Shanghai Jiao Tong University. The protocol was approved by the Human Ethics Committee of Ruijin Hospital, and written informed consents were obtained from the subjects. The Number in Human Ethics Committee is SIMMEC2022_122_Y. Baseline demographics and clinical characteristics of human subject cohorts are shown in Supplementary Table 1.

### miR-144 *in situ* hybridization with RNAscope probe

MiR-144 *in situ* hybridization was performed as previously reported 17, 18. In short, fresh tissues were embedded with OCT and sectioned at a thickness of 10 μm, and fixed overnight in pre-cooled 4% paraformaldehyde (PFA). After ethanol dehydration and protease IV (Advanced Cell Diagnostics, catalog 2018334, CA, USA) digestion, the sections were hybridized with an SR-RNU6 probe or SR-miR-144 probe (Advanced Cell Diagnostics) for two hours. Next, slides were conjugated to an alkaline phosphatase molecule (Advanced Cell Diagnostics, catalog 2018334, CA, USA) for chromogenic reactions. After each hybridization step, the slides were washed twice with 1 × PBS at room temperature for 2 minutes each. After blocking with 3% normal goat serum buffer for one hour at room temperature, the slides were incubated with the primary antibody (Desmin, Abcam, Cambridge, UK, catalog AB15200; Col1α1, Cell Signaling Technology, Danvers, MA, catalog 72026S) for 2 hours at room temperature. Then the anti-rabbit secondary antibody was applied to the slides for one hour at room temperature and the DAB Peroxidase Substrate Kit (Vector 2 Laboratories, catalog ZK1018, CA, USA) was used to visualize the staining according to the manufacturer's instructions. Nuclear staining was performed with hematoxylin (Servicebio, catalog CR2301055, Wuhan, China), and the slides were mounted with neutral balsam (MACKLIN, catalog C16129365, Shanghai, China). Images were obtained using the Olympus BX43 microscope or Olympus APEXVIEW APX100 microscope.

### Sirius red staining and Immunohistochemistry staining

For Sirius red staining, the paraffin-embedded sections were dewaxed in xylene and stained with PICRO-RED STAINING SOLUTION (Phygene, catalog 20230310, Fuzhou, China) for 1 hour. For immunohistochemistry, sections were subjected to antigen retrieval with citrate buffer (Invitrogen, catalog 005000, CA, USA). After incubating at 3% H_2_O_2_, sections were blocked with 3% normal goat serum buffer (NGS). Then the sections were incubated with primary antibodies overnight at 4°C and incubated with secondary antibodies (Cell Signaling Technology, Danvers, MA, catalog 8814S or 8125S) at room temperature for 1 hour. ImmPACT DAB Substrate Kit (Vector 2 Laboratories, catalog ZK1018, CA, USA) or ImmPACT Red Substrate Kit (Vector 2 Laboratories, catalog ZJ1205, CA, USA) were used to visualize the staining. Primary antibodies used were listed below: Desmin (Abcam, catalog AB15200, Cambridge, UK), Col1α1 (Cell Signaling Technology, catalog 72026S, Danvers, MA,), α-SMA (Cell Signaling Technology, catalog 19245S, Danvers, MA, USA; Thermo Fisher Scientific, catalog 14-9760-82, Waltham, MA, USA), Vimentin (Cell Signaling Technology, catalog 5741, Danvers, MA, USA), SIN3A (Thermo Fisher Scientific, catalog PA5-85554, Waltham, MA, USA), p-p38 (Cell Signaling Technology, catalog 4511, Danvers, MA, USA). Images were obtained using the Olympus BX43 microscope.

### Immunofluorescence staining

Fresh liver tissues were frozen and embedded for sectioning. After overnight fixation with 4% PFA at 4°C, 3% NGS was used to block for one hour at room temperature. Next, primary antibodies were incubated overnight at 4°C, and secondary antibodies (Alexa Fluor 488 or 555 goat anti-rabbit IgG [H+L], Cell Signaling Technology, catalog 4412S or 4413S; Alexa Fluor 488 or 555 goat anti-mouse IgG [H+L], Cell Signaling Technology, catalog 4408S or 4409S, Danvers, MA, USA) were incubated for 1 hour at room temperature. TrueVIEW Autofluorescence Quenching Kit (Vector 2 Laboratories, catalog ZK0818, CA, USA) was used to eliminate non-specific fluorescence. Nuclear staining was performed using 4', 6'-diamino-2-phenylindole (DAPI) (Beyotime, catalog P0131, Shanghai, China). Images were captured with Olympus APEXVIEW APX100 microscope.

### Total RNA isolation and reverse transcription quantitative PCR (RT-qPCR)

Total RNA was extracted using RNA isolater Total RNA Extraction Reagent (Vazyme, catalog 7E0131K3, Nanjing, China), and single-stranded cDNA was synthesized using the High-Capacity cDNA Reverse Transcription Kit (Thermo Fisher Scientific, catalog 2816898, Waltham, MA). Gene expression was determined by qPCR using ChamQ Universal SYBR qPCR Master Mix (Vazyme, catalog 7E751K3, Nanjing, China) and a QuantStudio 5 Instrument (Thermo Fisher Scientific, Waltham, MA). The mRNA levels of *18s* were used as an internal control. Each test was performed in triple replication and the 2^-∆∆Ct^ method was used to calculate the expression of mRNA. The primers used for RT-qPCR are listed in Supplementary Table 2.

For miRNA detection, the mature miRNA strand cDNA was synthesized using miRNA 1st Strand cDNA Synthesis Kit (Vazyme, catalog 7E761E3, Nanjing, China) and amplified using miRNA Universal SYBR qPCR Master Mix (Vazyme, catalog MQ101-02-AA, Nanjing, China). The fold-change for miRNA relative to snoRNA202, RUN44 was determined by the formula 2^-∆∆Ct^. The primers used for miRNA detection are listed in Supplementary Table 3.

### Western blotting

Proteins were extracted from liver tissues or cells using RIPA Buffer (Thermo Fisher Scientific, catalog YH374135, Waltham, MA, USA) containing Halt Protease and Phosphatase Inhibitors (Thermo Fisher Scientific, catalog 78447, Waltham, MA, USA). The samples were loaded into polyacrylamide gels (Absin, catalog 220A019, Shanghai, China) and then transferred onto nitrocellulose membranes (Merck, catalog 0000208128, Darmstadt, Germany). The nitrocellulose membranes were blocked with 1% BSA and incubated with antibodies overnight at 4°C. After incubation with anti-rabbit or anti-mouse IgG HRP-linked second antibody (Cell Signaling Technology, catalog 7074S or 7076S, Danvers, MA, USA), protein bands were visualized with SuperSignal Maximum Sensitivity Substrate (Thermo Fisher Scientific, catalog WG328673, Waltham, MA, USA). The following antibodies were used: Col1α1 (Abcam, Cambridge, UK, catalog AB260043), α-SMA (Thermo Fisher Scientific, catalog 14-9760-82, Waltham, MA, USA), Vimentin (Cell Signaling Technology, Danvers, MA, catalog 5741), SIN3A (ABclonal Technology, catalog A1577, Wuhan, China), p38 (Cell Signaling Technology, catalog 8690S, Danvers, MA, USA), p-p38 (Cell Signaling Technology, catalog 4511, Danvers, MA, USA), ERK (Cell Signaling Technology, catalog 4695S, Danvers, MA, USA), p-ERK (Cell Signaling Technology, catalog 4370S, Danvers, MA, USA), p-Smad2/3 (Cell Signaling Technology, catalog 8828S, Danvers, MA, USA), Smad2/3 (Cell Signaling Technology, catalog 8685S, Danvers, MA, USA).

### Isolation of primary HSCs

Mouse primary HSCs were isolated as previously described 19. Briefly, after the mice were anesthetized with pentobarbital, the inferior vena cava was intubated, and then the portal vein was cut and the superior vena cava was clamped with vascular clips. Murine livers were sequentially perfused with Ethylene glycol tetraacetic acid (EGTA), pronase solutions (Merck, catalog P5147, Darmstadt, Germany), and collagenase solutions (Worthington, catalog 41J21479, CO, USA). After *in vitro* digestion for 30 minutes, hepatocytes were separated by centrifugation at 400 rpm for 5 minutes, and the supernatant was centrifuged at 1600 rpm for 10 minutes to collect the precipitate. After centrifugation, the cell pellet was suspended in 20% OptiPrep (Merck, catalog D1556, Darmstadt, Germany) and carefully added 11.5% OptiPrep and Hank's Balanced Salt Solution (HBSS) respectively on it. After centrifugation at 3000 rpm for 17 minutes at 4°C, the cell fraction between 11.5% OptiPrep and HBSS solution was collected gently and washed with HBSS solution. Eventually, primary HSCs were harvested and cultured in RPMI-1640 medium containing 10% fetal bovine serum and 1% penicillin-streptomycin.

### Cell culture and transfection of miR-144 mimics

Human hepatic stellate cell line LX-2 was a kind gift from Prof. Chen Jing, Shanghai Institute of Materia Medica (SIMM), Chinese Academy of Sciences. Primary HSCs and LX-2 cells were cultured in RPMI 1640 medium (Gibco, catalog 2786824, NY, USA) containing 10% fetal bovine serum (Gibco, catalog 2500251P, NY, USA), and penicillin-streptomycin (Gibco, catalog 15140-122, NY, USA). Mouse hepatic stellate cell line JS-1 was a kind gift from Prof. Man Li, Shuguang Hospital Affiliated to Shanghai University of Traditional Chinese Medicine. JS-1 cells were cultured in DMEM medium (Corning, catalog 31222002, NY, USA) containing 10% fetal bovine serum (Gibco, catalog 2500251P, NY, USA), and penicillin-stretomycin (Gibco, catalog 15140-122, NY, USA).

The transfection of miRNA mimics was performed in primary HSCs, LX-2 cells, and JS-1 cells. In brief, negative control mimics (NC mimics) or miR-144 mimics (Sangon, Shanghai, China) were transfected into cells at 50 nM using Lipofectamine RNAiMAX transfection reagent (Invitrogen, catalog 2487720, CA, USA). Total RNA or protein was extracted after transfection for 48 hours.

### Measurement of hepatic hydrogen peroxide (H_2_O_2_) content

H_2_O_2_ content was determined using the H_2_O_2_ Assay Kit (Beyotime, catalog 052323231207, Shanghai, China). Frozen liver tissues were lysed by H_2_O_2_ Lysis Buffer according to the manufacturer's instruction.

### Dual-luciferase activity assay

HEK293 cell line was cotransfected with control luciferase vector (100 ng) (WZ Biosciences, Shandong, China), Sin3a 3'-untranslated region (UTR) vector (100 ng) (WZ Biosciences, Shandong, China), and miR-144 mimics or NC-mimics by Lipofectamine™ 3000 (Invitrogen, catalog CN2498094, CA, USA) for 24 hours. The Firefly and Renilla luciferase activities were measured by using the Duo-Lite Luciferase Assay System (Vazyme, catalog 1205E705, Nanjing, China) 24h after transfection according to the manufacturer's instruction. The luminescence amount of firefly luciferase compared to Renilla luciferase is calculated as relative luciferase activity.

### Biochemical assays

Serum alanine aminotransferase (ALT) and aspartate aminotransferase (AST) levels were determined using ALT Detection Kit (Nanjing Jiancheng Bioengeering Institute, China, catalog C009-3-1) and AST Detection Kit (Nanjing Jiancheng Bioengeering Institute, China, catalog C010-3-1) according to the manufacturer's instruction.

### Statistical analysis

Data are expressed as the means ± SEM and were analyzed using GraphPad Prism software (version 9.4.1; GraphPad Software). To compare values obtained from two groups, the Student *t* test was performed; values from multiple groups were compared using one-way ANOVA. *P* < 0.05 was considered significant.

## Results

### Oxidative stress significantly elevates the expression of miR-144, which promotes HSC activation

**Figure 1:**
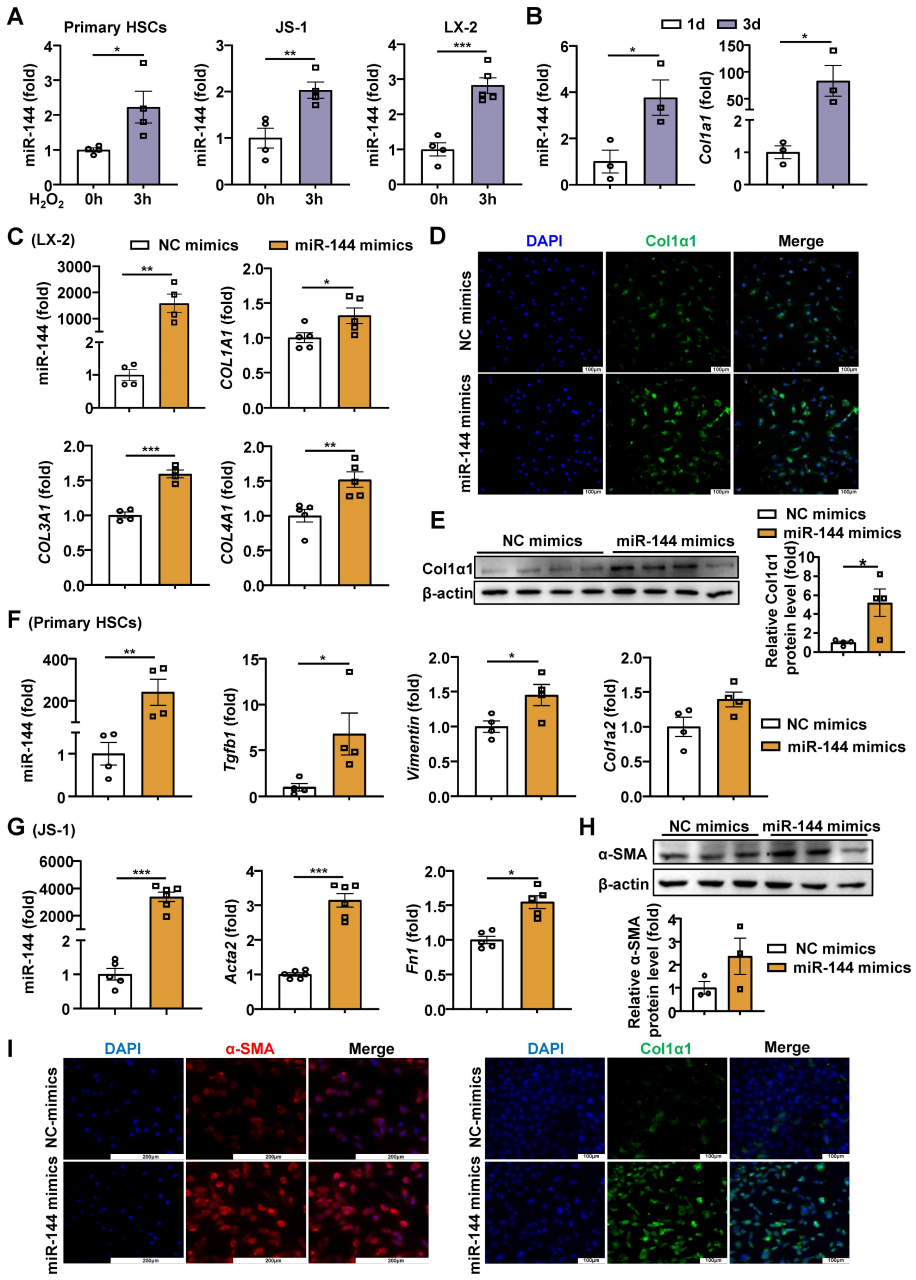
** Oxidative stress-driven miR-144 promotes HSC activation *in vitro*** (A) HSCs were treated with H_2_O_2_ (200 μM) or vehicle for 3 hours and subjected to miR-144 analysis. (B) Primary HSCs were isolated and cultured for 1 day and 3 days, the expression of *Col1a1* and miR-144 were analyzed by RT-qPCR. (C-E) LX-2 cell lines were transfected with miR-144 mimics and NC mimics for 48 hours. The expression of miR-144, *COL1A1*, *COL3A1*, and *COL4A1* were analyzed by RT-qPCR in panel C. Representative images of Col1α1 (green) and nuclei (blue) are shown in panel D. Scale bars: 100 μm. Col1α1 expression was analyzed by Western blot and quantified in panel E. (F) Primary HSCs were transfected with miR-144 mimics and NC mimics for 48 hours. The expression of miR-144, *Col1a2*, *Vimentin*, and *Tgfb1* were analyzed by RT-qPCR. (G-I) JS-1 cell lines were transfected with miR-144 mimics and NC mimics for 48 hours. The expression of miR-144, *Acta2*, and *FN1* were analyzed by RT-qPCR analyses in panel G. α-SMA expression was analyzed by Western blot in panel H. Representative images of α-SMA staining (red) (Scale bars: 200 μm), Col1α1 staining (green) (Scale bars: 200 μm), and nuclei (blue) are shown in panel I. Values represent means ± SEM from three independent experiments. *P < 0.05, **P < 0.01, ***P < 0.001.

Previous studies have shown that the H_2_O_2_ content is elevated in liver of mice with CCl_4_-induced liver fibrosis 20. To further confirm whether hepatic oxidative stress during liver fibrosis, we detected the H_2_O_2_ content in mice with CCl_4_-induced liver fibrosis. Unexpectedly, H_2_O_2_ content was not elevated significantly (Supporting Fig. S1A), possibly because in this model mice were sacrificed 48 hours after the last dose of CCl_4_, when H_2_O_2_ levels may return to the basal level. Therefore, we examined the H_2_O_2_ content in acute CCl_4_ model and found that hepatic H_2_O_2_ in mice administered CCl_4_ was significantly elevated compared with the control group (Supporting Fig. S1B). In proportion, we measured the expression of oxidant related genes in the liver and found that the expression of *Ho-1* and *Nqo-1* was significantly up-regulated in mice with CCl_4_-induced liver fibrosis (Supporting Fig. S1C). Next, we also detected the oxidative stress in BDL models, and found the hepatic H_2_O_2_ content and the mRNA levels of *Ho-1* and *Nqo-1* were significantly elevated (Supporting Fig. S1D-E). To investigate whether oxidative stress affected miR-144 in HSCs *in vitro*, we measured miR-144 in HSCs after treatment with H_2_O_2_, which is the experimental condition that produces abundant oxidative stress 21, 22. Intriguingly, H_2_O_2_ significantly elevated miR-144 in mouse primary HSCs, mouse HSC cell line JS-1 cells and human HSC cell line LX-2 cells (Fig. 1A). However, buthionine-sulfoximine (BSO), another experimental condition to produce oxidative stress, didn't affect the expression of miR-144 in HSCs (Supporting Fig. S2A). Although the TGF-β stimulation did not increase miR-144 expression in HSCs (Supporting Fig. S2B), the expression of miR-144 in mouse primary HSCs was altered during HSC activation* in vitro* as demonstrated that the expression of miR-144 was markedly upregulated in activated HSCs on day 3 compared to quiescent HSCs (Fig. 1B). Next, we wondered whether miR-144 affects HSC activation. As illustrated in Fig. 1C-E, overexpression of miR-144 by miRNA mimics was performed in LX-2 cells, and the transfection efficiency was validated. Importantly, overexpression of miR-144 exerted an identical pro-fibrogenic effect as indicated by significantly elevated *COL1A1*, *COL3A1*, and *COL4A1* mRNA levels and COL1α1 protein levels. Overexpression of miR-144 in primary HSCs also upregulated the mRNA levels of fibrogenic genes such as *Col1a2, Vimentin,* and *Tgfb1* (Fig. 1F). In parallel, we obtained the consistent results by transfecting miR-144 mimics in the JS-1 cells (Fig. 1G-I). Furthermore, inhibition of miR-144 by miR-144 inhibitor was performed in LX-2 cells. The mechanism of miR-144 inhibitor relies on the complementary base pairing of the oligonucleotide sequence to miR-144, which may not lead to the downregulation of miR-144 23, 24. Blockage of miR-144 repressed the expression of fibrogenic genes including *ACTA2*, *COL3A1* and *FN1* (Supporting Fig. S3A-B). All the data suggest that miR-144 may play an important role in the activation of HSCs. Besides, we wondered whether oxidative stress regulates the expression of miR-451 (the miR-144 cluster neighbor). Administration of H_2_O_2_ or BSO slightly reduced the expression of miR-451 in HSCs (Supporting Fig. S4A). Interestingly, overexpression of miR-451 in LX-2 cells did not affect HSC activation (Supporting Fig. S4B).

### MiR-144 is preferentially elevated in HSCs of mice with CCl_4_ and BDL-induced liver fibrosis

**Figure 2:**
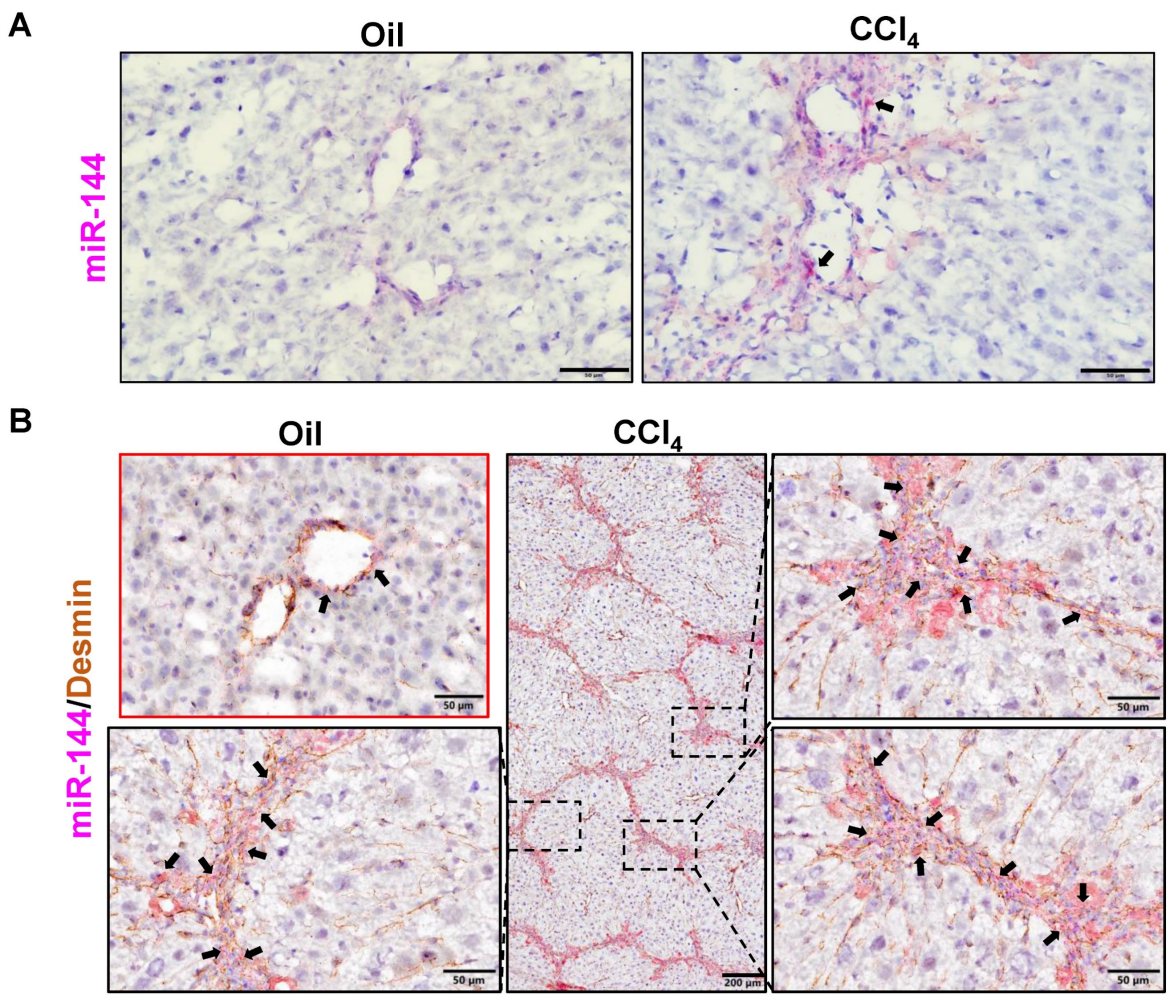
** MiR-144 is preferentially elevated in HSCs of mice with CCl_4_-induced liver fibrosis.** C57BL/6J mice were injected intraperitoneally with 2 ml/kg weight of 10% CCl_4_ in olive oil twice a week for 6 weeks. Liver tissue samples were collected. (A) Frozen liver tissue sections were subjected to miR-144 *in situ* hybridization with RNAscope probe. Representative images of miR-144 expression are shown. Black arrows indicate miR-144^+^ areas. Scale bars: 50 μm. (B) Frozen liver tissue sections were subjected to miR-144 *in situ* hybridization with RNAscope along with immunohistochemistry staining of HSC marker Desmin. Representative images of miR-144 expression (red), Desmin (brown), and nuclei (blue) are shown. Black arrows indicate miR-144^+^ HSCs. Scale bars: 200 μm or 50 μm.

**Figure 3:**
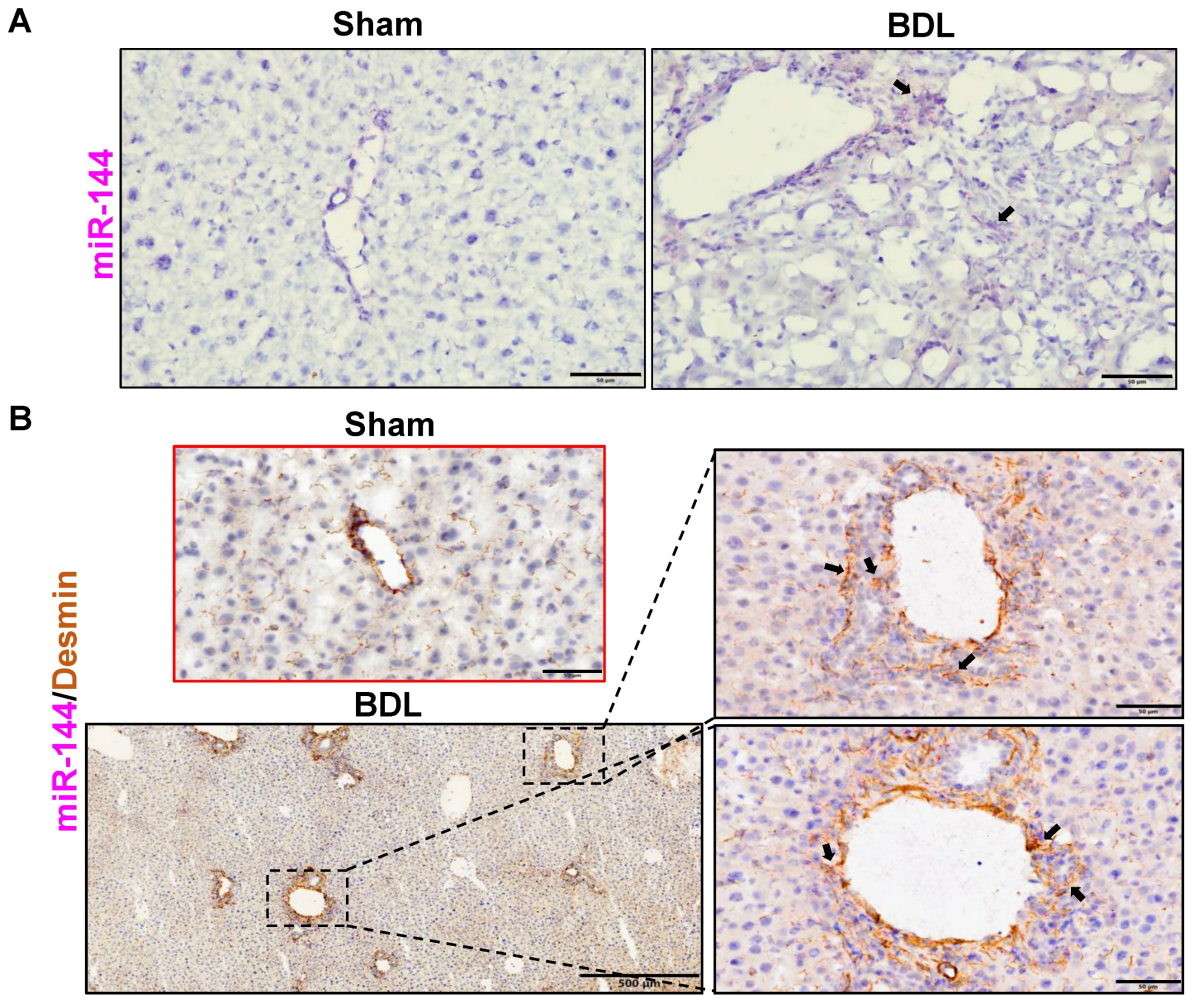
** MiR-144 is also specifically observed in HSCs of mice with BDL-induced liver fibrosis.** C57BL/6J mice were performed BDL operation for 2 weeks. Liver tissue samples were collected. (A) Frozen liver tissue sections were subjected to miR-144 *in situ* hybridization with RNAscope probe. Representative images of miR-144 expression are shown. Black arrows indicate miR-144^+^ areas. Scale bars: 50 μm. (B) Frozen liver tissue sections were subjected to miR-144 *in situ* hybridization with RNAscope along with immunohistochemistry staining of HSC marker Desmin. Representative images of miR-144 expression (red), Desmin (brown), and nuclei (blue) are shown. Black arrows indicate miR-144^+^ HSCs. Scale bars: 500 μm or 50 μm.

To investigate the role of miR-144 in the development of liver fibrosis, we first performed *in situ* hybridization analyses to detect hepatic miR-144 expression in mice with CCl_4_ and BDL-induced liver fibrosis, which are two well-established liver fibrosis mouse models. Remarkably, the expression of miR-144 was extremely elevated in the liver of mice with CCl_4_-induced liver fibrosis (Fig. 2A). Moreover, since miR-144 prominently covered fibrotic areas, we hypothesized that miR-144 might be exclusively expressed in HSCs during liver fibrosis. To characterize the major cell type expressing miR-144, we performed *in situ* hybridization analyses and immunohistochemical staining of the HSC marker Desmin. Consistent with the assumption, miR-144 was preferentially detected in HSCs but not hepatocytes or cholangiocytes in liver samples from mice with CCl_4_-induced liver fibrosis (Fig. 2B and Supporting Fig. S5A). Next, we detected miR-144 expression in BDL-induced liver fibrosis model. As expected, miR-144 was also specifically observed in substantial HSCs in mice with BDL-induced liver fibrosis (Fig. 3A-B and Supporting Fig. S5B).

### Administration of AAV6-miR-144 antagomir alleviates CCl_4_-induced liver fibrosis

**Figure 4:**
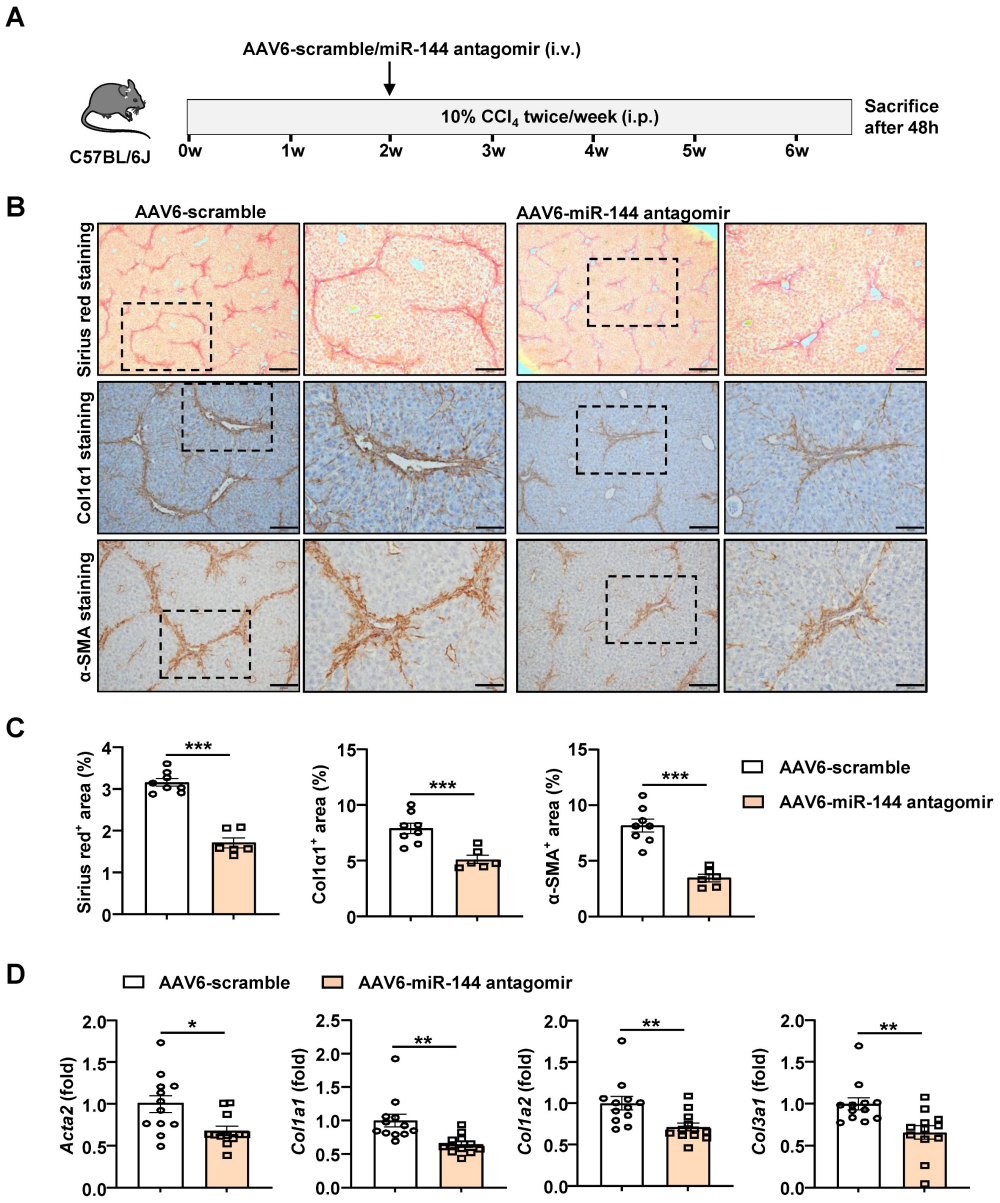
** Blockade of miR-144 in HSCs significantly alleviated CCl_4_-induced liver fibrosis.** (A) Schematic overview of the experimental design. Liver tissue samples were collected. (B) Representative images of Sirius red staining (Scale bars: 500 μm or 200 μm), Col1α1 staining (Scale bars: 200 μm or 100 μm), and α-SMA staining (Scale bars: 200 μm or 100 μm) of liver tissue sections are shown. (C) Quantification of fibrotic area per field. (D) RT-qPCR analyses of hepatic fibrogenesis genes. Values represent means ± SEM (n=12 per group). *P < 0.05, **P < 0.01, ***P < 0.001.

Based on the above evidence that miR-144 promotes the activation of HSCs *in vitro*, we speculated that miR-144 might accelerate the progression of liver fibrosis *in vivo*. In support of this notion, we intended to generate HSC-specific miR-144 knockout mice (LRAT-Cre, miR-144^fl/fl^) to explore its function. Unfortunately, miR-144^fl/fl^ mice could not be constructed due to the proximity of miR-144 and the isogenic cluster miR-451. However, emerging evidence revealed that Adeno-Associated Virus 6 (AAV6) exhibits organ tropism for activated HSCs in mice 25, 26. To further clarify the infection efficiency of AAV6 on HSCs, mice were administrated with AAV6-green fluorescent protein (GFP) through tail vein injection. Then we performed double immunofluorescence staining of hepatic GFP and Desmin and indeed found AAV6 markedly and exclusively infected HSCs (Supporting Fig. S6). To suppress the function of miR-144, we combined AAV6 vector with miR-144 “tough decoys” to specifically and persistently inhibit miR-144 in CCl_4_-induced liver fibrosis due to that Tough Decoy-RNA (Tud-RNA) is a well-known double-stranded stem-loop structure to inhibit specific miRNAs in mammalian cells 27, 28 (Fig. 4A). As shown in Fig. 4B-C, Sirius red staining, α-SMA staining, and Col1α1 staining suggested that mice given AAV6-miR-144 antagomir had lower degree of liver fibrosis compared with mice given AAV6-scramble. Furthermore, the expression of several fibrogenic genes including *Acta2, Col1a1, Col1a2,* and *Col3a1* was significantly suppressed after administration of AAV6-miR144 antagomir (Fig. 4D). However, blockade of miR-144 in HSCs did not affect liver injury (Supporting Fig. S7). The above results strongly suggest that specific inhibition of miR-144 in HSCs mitigates CCl_4_-induced liver fibrosis.

### Mice administrated of AAV6-miR-144 agomir are more susceptible to CCl_4_-induced liver fibrosis

**Figure 5:**
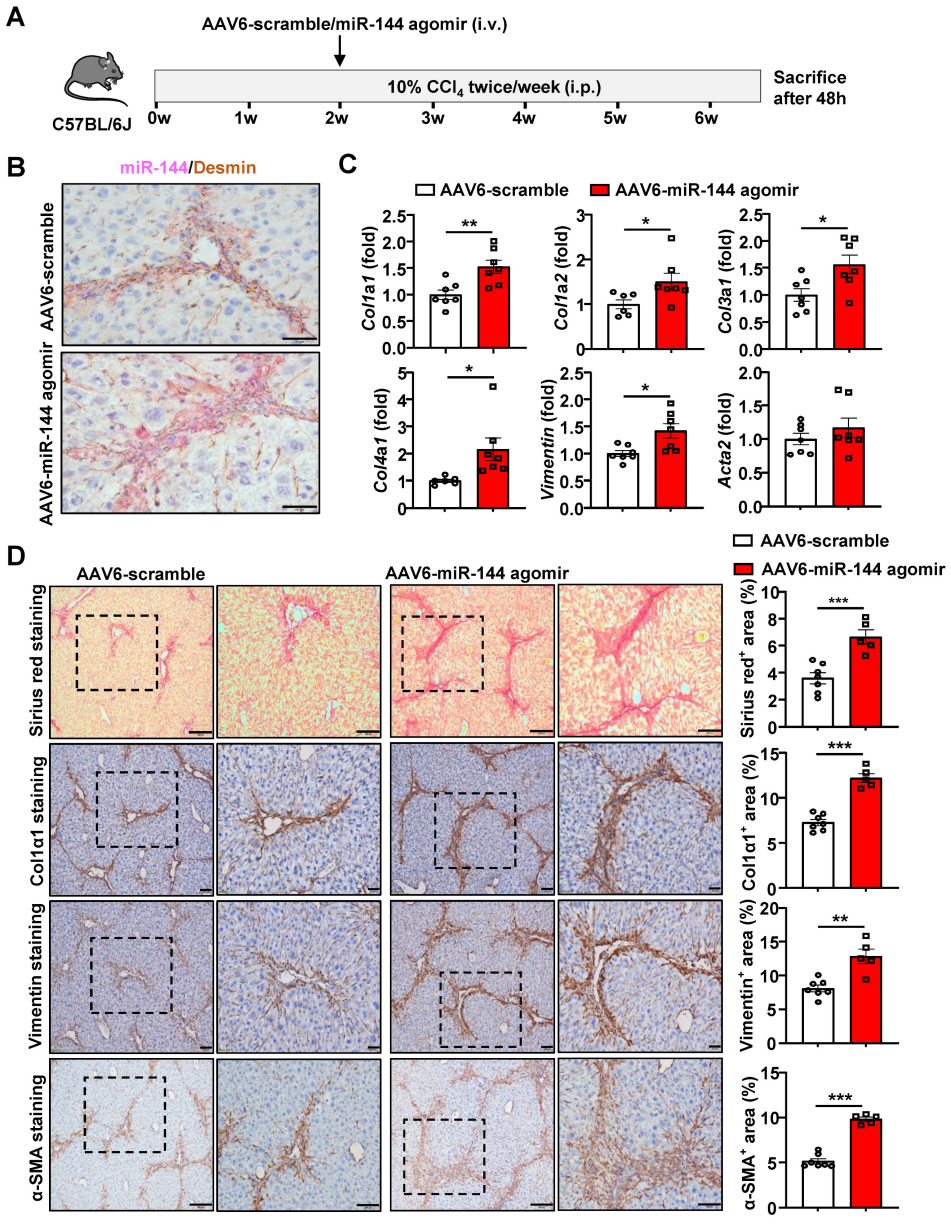
** Overexpression of miR-144 in HSCs accelerated the progression of CCl_4_-induced liver fibrosis.** (A) Schematic overview of the experimental design. Liver tissue samples were collected. (B) Frozen liver tissue sections were subjected to miR-144 *in situ* hybridization with RNAscope along with immunohistochemistry staining of HSC marker Desmin. Representative images of miR-144 expression (red), Desmin (brown), and nuclei (blue) are shown. Scale bars: 50 μm. (C) RT-qPCR analyses of several hepatic fibrogenesis genes. (D) Representative images of Sirius red staining (Scale bars: 200 μm or 100 μm), Col1α1 staining (Scale bars: 100 μm or 50 μm), Vimentin staining (Scale bars: 100 μm or 50 μm) and α-SMA staining (Scale bars: 200 μm or 100 μm) of liver tissue sections are shown. Quantification of fibrotic area per field. Values represent means ± SEM (n=7 per group). *P < 0.05, **P < 0.01, ***P < 0.001.

To further validate the pro-fibrotic role of miR-144 *in vivo*, we specifically overexpressed miR-144 in HSCs of CCl_4_-induced mouse fibrosis model by injection with AAV6-miR-144 agomir, which integrates miR-144 mimics into AAV6 vector (Fig. 5A). First, we verified that miR-144 was indeed overexpressed in HSCs by *in situ* hybridization and RT-qPCR as shown in Fig. 5B and Supporting Fig. S8A. RT-qPCR analyses demonstrated that the expression of fibrogenic genes (*Col1a1, Col1a2, Col3a1, Col4a1,* and *Vimentin*) was significantly augmented upon AAV6-miR-144 agomir treatment except *Acta2* (Fig. 5C). Consistently, accelerated liver fibrosis development was identified by Sirius Red staining, α-SMA staining, Col1α1 staining, and Vimentin staining after overexpression of miR-144 in HSCs (Fig. 5D). Interestingly, although there was no difference in the expression of fibrogenic genes, Sirius red staining, Col1α1 staining, and western blot results demonstrated that administration of miR-144 agomir slightly facilitated liver fibrosis in mice injected with olive oil (Supporting Fig. S9A-D). Although miR-144 promoted the progression of liver fibrosis, miR-144 reduced CCl_4_-induced liver injury (Supporting Fig. S8B). Taken together, these data strongly suggest that miR-144 exerts a pro-fibrotic effect in the development of liver fibrosis *in vivo*.

### MiR-144 aggravates BDL-induced liver fibrosis

**Figure 6:**
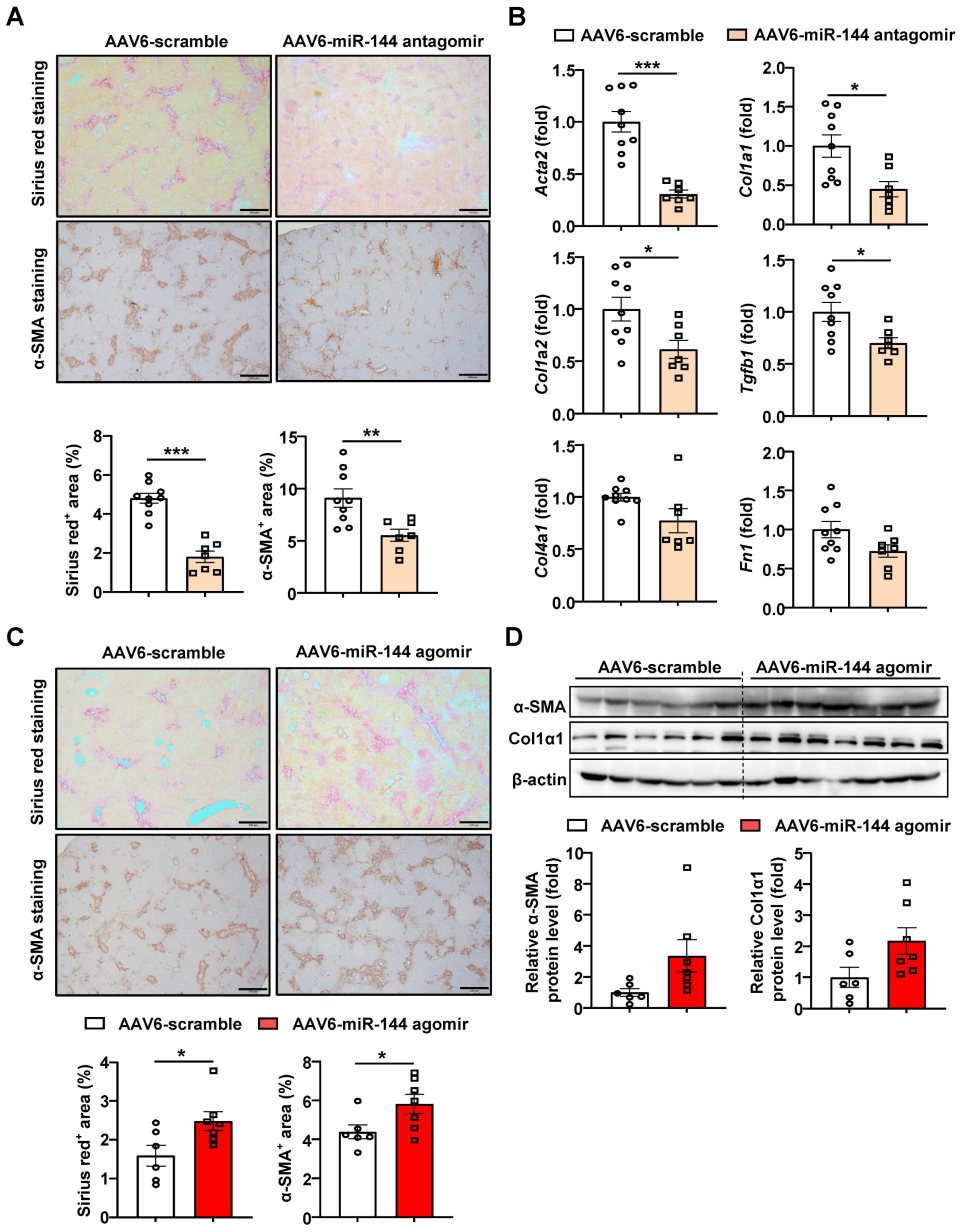
** Inhibition of miR-144 in HSCs ameliorated, overexpression of miR-144 aggravated BDL-induced liver fibrosis.** (A-D) C57BL/6J mice were performed BDL operation for 17 days. AAV6-scramble, AAV6-miR-144 antagomir, and AAV6-miR-144 agomir were administered to mice via the tail vein three days after surgery. (A, C) Representative images of Sirius red staining (Scale bars: 500 μm) and α-SMA staining (Scale bars: 500 μm) of liver tissue sections are shown. Quantification of fibrotic area per field. (B) RT-qPCR analyses of several hepatic fibrogenesis genes. (D) Western blot analyses of α-SMA and Col1α1 expression. Values represent means ± SEM (n=7-9 per group). *P < 0.05, **P < 0.01, ***P < 0.001.

Next, miR-144 antagomir/agomir was administered to BDL-induced liver fibrosis mice three days after surgery. Intriguingly, blocking miR-144 alleviated the extent of BDL-induced liver fibrosis, as shown by Sirius red staining and a-SMA staining (Fig. 6A). In concordance, mice administrated miR-144 antagomir exhibited lower fibrogenic gene expression, such as *Acta2, Col1a1, Col1a2, Tgfb1, Col4a1, and Fn1* (Fig. 6B). Consistently, administration of AAV6-miR-144 agomir significantly elevated collagen and a-SMA protein expression as illustrated by Sirius red and a-SMA staining results although it didn't reach statistical significance by western blot results (Fig. 6C-D), suggesting that miR-144 may also exacerbate BDL-induced liver fibrosis. Besides, miR-144 did not affect BDL-induced liver injury (Supporting Fig. S10A-B).

### MiR-144 drives HSC activation by limiting SIN3A-p38 axis

**Figure 7:**
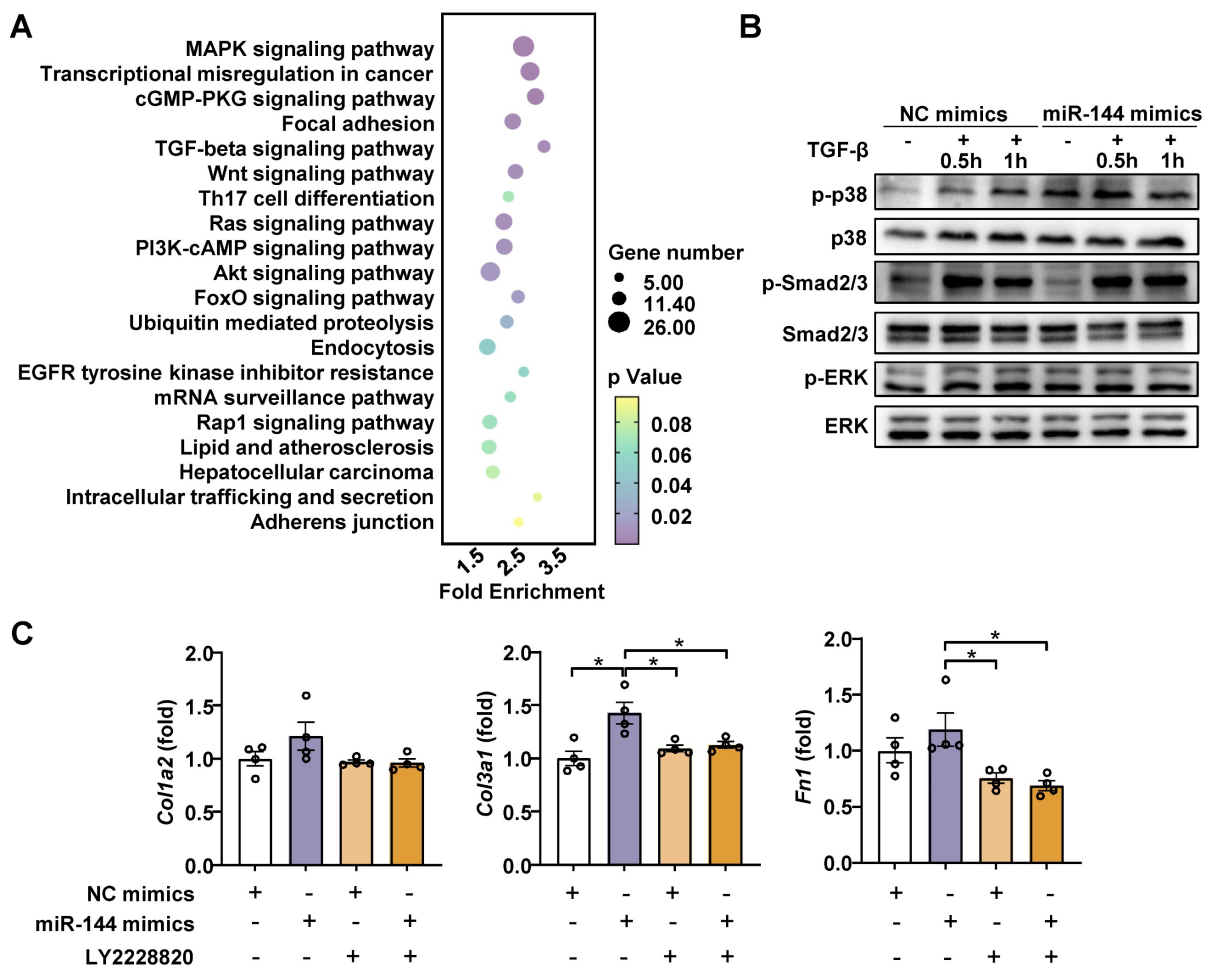
** MiR-144 promotes p38 activation in HSCs.** (A) The GO and KEGG enrichment analysis of all miR-144 predicted target genes. Selected significant signaling pathways were shown in bubble diagram. (B) LX-2 cell lines were transfected with miR-144 mimics and NC mimics for 48 hours and then treated with TGF-β (10 ng/ml) for the indicated time points. The p-p38 and p-Smad2/3 proteins were measured by western blotting. (C) LX-2 cell lines were transfected with miR-144 mimics and NC mimics for 24 hours and then treated with p38 inhibitor LY2228820 (2 μM) for 24 hours. RT-qPCR analyses of *Col1a2*, *Col3a1*, and *Fn1* expression. Values represent means ± SEM from three independent experiments. *P < 0.05, **P < 0.01.

Generally, miRNAs have multiple target genes and play a regulatory role by inhibiting the translation of mRNA 29. To elucidate the mechanism by which miR-144 promotes liver fibrosis progression, the enrichment of miR-144 predicted target genes (TargetScan, https://www.targetscan.org/mmu_80/) was determined by Gene Ontology (GO) and Kyoto Encyclopedia of Genes and Genomes (KEGG) pathway analyses. The analyses revealed that multiple predicted target genes were enriched in MAPK and TGF-beta signaling pathways (Fig. 7A), which are classic signaling pathways contributing to the development and progression of liver fibrosis 30, 31. To validate our observation, we examined which signaling pathway is involved in the effect of miR-144 on liver fibrosis. As illustrated in Fig. 7B, overexpression of miR-144 exerted more p38 activation (p-p38) during TGF-β1 stimulation, but not Smad2/3 or ERK activation, in LX-2 cell line. Importantly, p38 inhibitor (LY2228820) treatment blocked the induction of *Col1a2, Col3a1,* and* Fn1* by miR-144 in HSCs (Fig. 7C).

**Figure 8:**
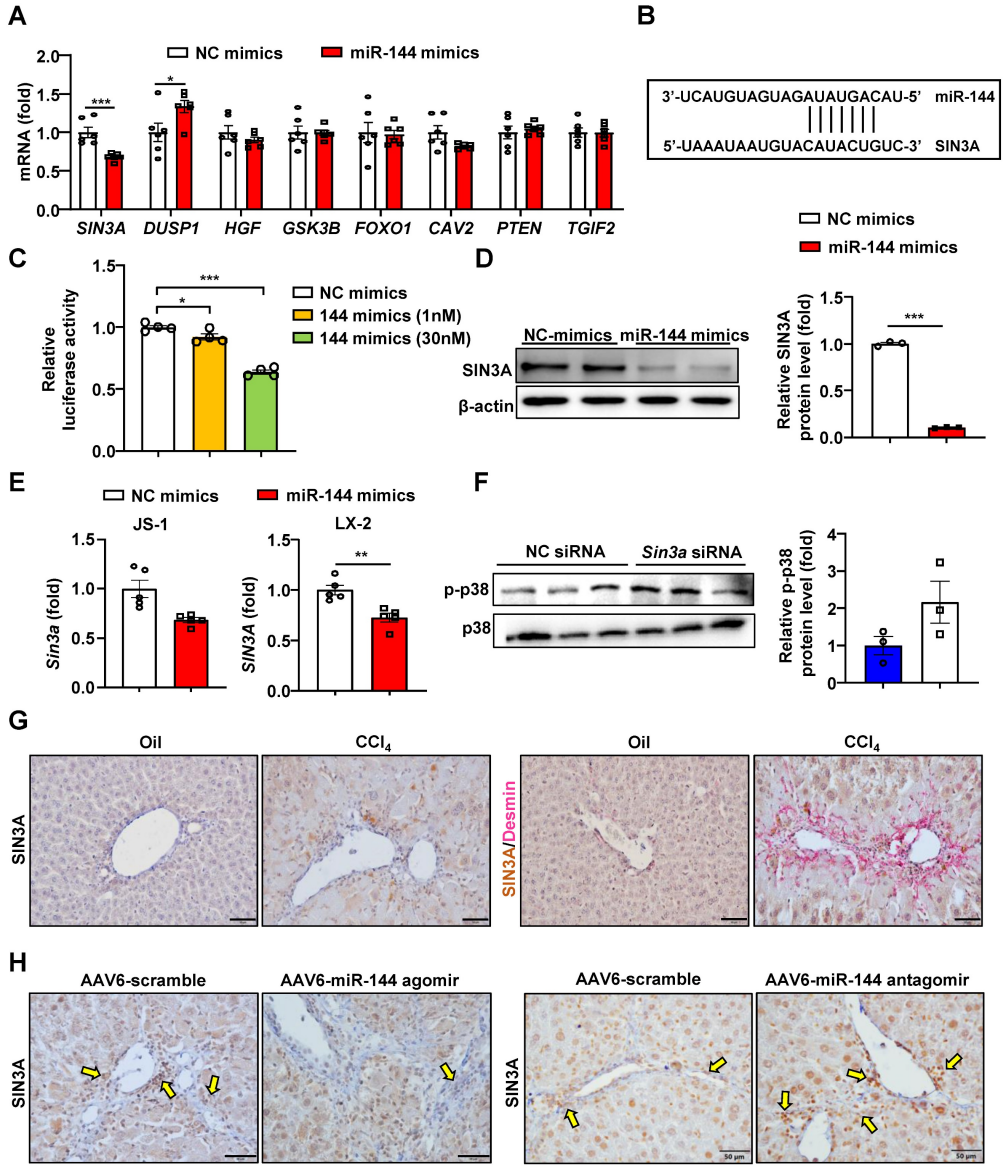
** MiR-144 controls SIN3A-p38 axis in HSCs.** (A) LX-2 cell lines were transfected with miR-144 mimics and NC mimics for 48 hours. RT-qPCR analyses of several miR-144 potential target genes. (B) Bioinformatics approach analysis of the target prediction of miR-144. (C) Dual-luciferase activity assay was performed to verify the binding between miR-144 and the 3'-UTR of *Sin3a*. HEK293 cells were cotransfected with Sin3a 3'-UTR vector (100 ng), miR-144 mimics or NC-mimics for 24 hours. Relative luciferase activity was determined. (D) Western blot analysis and quantification of SIN3A expression in LX-2 cell lines after transfection of miR-144 mimics. (E) RT-qPCR analysis of *Sin3a* expression in JS-1 and LX-2 cell lines after transfection of miR-144 mimics. (F) JS-1 cell lines were transfected with Sin3a siRNA or NC siRNA for 48 hours. Protein levels were determined by western blot analyses. (G) C57BL/6J mice were injected intraperitoneally with 2 ml/kg weight of 10% CCl_4_ in olive oil twice a week for 6 weeks. Representative images of SIN3A and Desmin double staining of liver tissue sections are shown. Scale bars: 50 μm. (H) Liver of mice administrated of AAV6-scramble, AAV6-miR-144 antagomir, and AAV6-miR-144 agomir were subjected to SIN3A immunohistochemistry staining. Arrows indicate SIN3A^+^ areas. Values represent means ± SEM from three independent experiments. Scale bars: 50 μm.*P < 0.05, **P < 0.01, ***P < 0.001.

Next, we intended to further explore the downstream regulatory mechanism of miR-144. The prediction of the miR-144 target genes was performed by TargetScan, and found some potential target genes of miR-144 (Fig. 8A-B). Finally, we paid more attention to SIN3A, which may play an important role in profibrotic effects 32. Overexpression of miR-144 in HEK293 cells significantly reduced luciferase activity from the Sin3a 3'-UTR vector, suggesting that *Sin3a* is the target of miR-144 (Fig. 8C). More importantly, transfection of miR-144 mimics suppressed the expression of SIN3A at the mRNA and protein levels in HSCs (Fig. 8D-E). Besides, silencing of *Sin3a* obviously promoted p38 activation (Fig. 8F). Our findings further confirmed that SIN3A expression was upregulated in Desmin^+^ HSCs (Fig. 8G). Moreover, miR-144 targeting SIN3A was also corroborated in mice with CCl_4_-induced liver fibrosis, which were administrated with AAV6-miR-144 agomir/antagomir. As illustrated in Fig. 8H, overexpression of miR-144 in HSCs decreased SIN3A expression, whereas inhibition of miR-144 obviously elevated its expression in CCl_4_-induced liver fibrosis.

### Specific knockdown of *Sin3a* in HSCs exacerbates liver fibrosis

**Figure 9:**
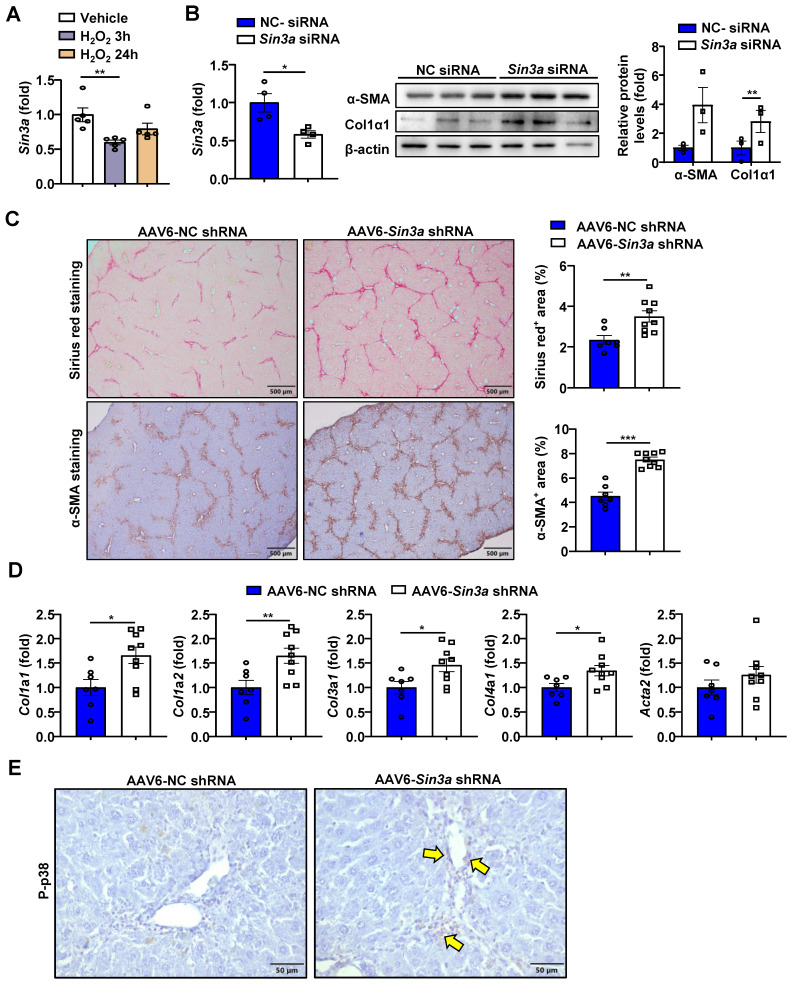
** Specific knockdown of *Sin3a* in HSCs exacerbates liver fibrosis.** (A) JS-1 cell lines were treated with H_2_O_2_ (200 μM) for the indicated time points. The *Sin3a* mRNA expression was measured by RT-qPCR. (B) JS-1 cells were transfected with *Sin3a* siRNA and NC siRNA for 48 hours. RT-qPCR analyses of *Sin3a* mRNA and western blotting analyses of fibrosis-related proteins. (C-E) C57BL/6J mice were injected intraperitoneally with 2 ml/kg weight of 10% CCl_4_ in olive oil twice a week CCl_4_ for 2 weeks before administering with AAV6-*Sin3a* shRNA. After AAV6 injection, another 8 doses of CCl_4_ were administered to the mice, which were sacrificed 48 hours after the last injection of CCl_4_ (n=7-9). (C) Liver tissues were subjected to Sirius red staining (Scale bars: 500 μm) and α-SMA staining (Scale bars: 500 μm). Quantification of fibrotic area per field. (D) RT-qPCR analyses of several hepatic fibrogenesis genes. (E) Representative images of p-p38 staining of liver tissue sections are shown. Yellow arrows indicate p-p38^+^ areas. Scale bars: 50 μm. Values represent means ± SEM from three independent experiments for *in vitro* experiment. *P < 0.05, **P < 0.01.

As indicated above, H_2_O_2_ elevates miR-144 expression in HSCs. Therefore, we reasoned that such elevation may inhibit SIN3A expression. Indeed, we found that H_2_O_2_ treatment decreased the expression of *Sin3a* mRNA in JS-1 cell lines (Fig. 9A). To elucidate whether SINA3A was involved in the progression of liver fibrosis, we firstly transfected *Sin3a* siRNA in JS-1 cell lines. As illustrated in Fig. 9B, knockdown of *Sin3a* resulted in a similar exacerbated fibrogenic effect as well as overexpression of miR-144 *in vitro*. In proportion, injection of AAV6-*Sin3a* shRNA remarkably aggravated the degree of liver fibrosis as demonstrated by Sirius red staining and a-SMA staining (Fig. 9C). Meanwhile, knockdown of *Sin3a* in HSCs also promoted the expression of fibrogenic genes at the mRNA levels and p38 activation (Fig. 9D and E).

**Figure 10:**
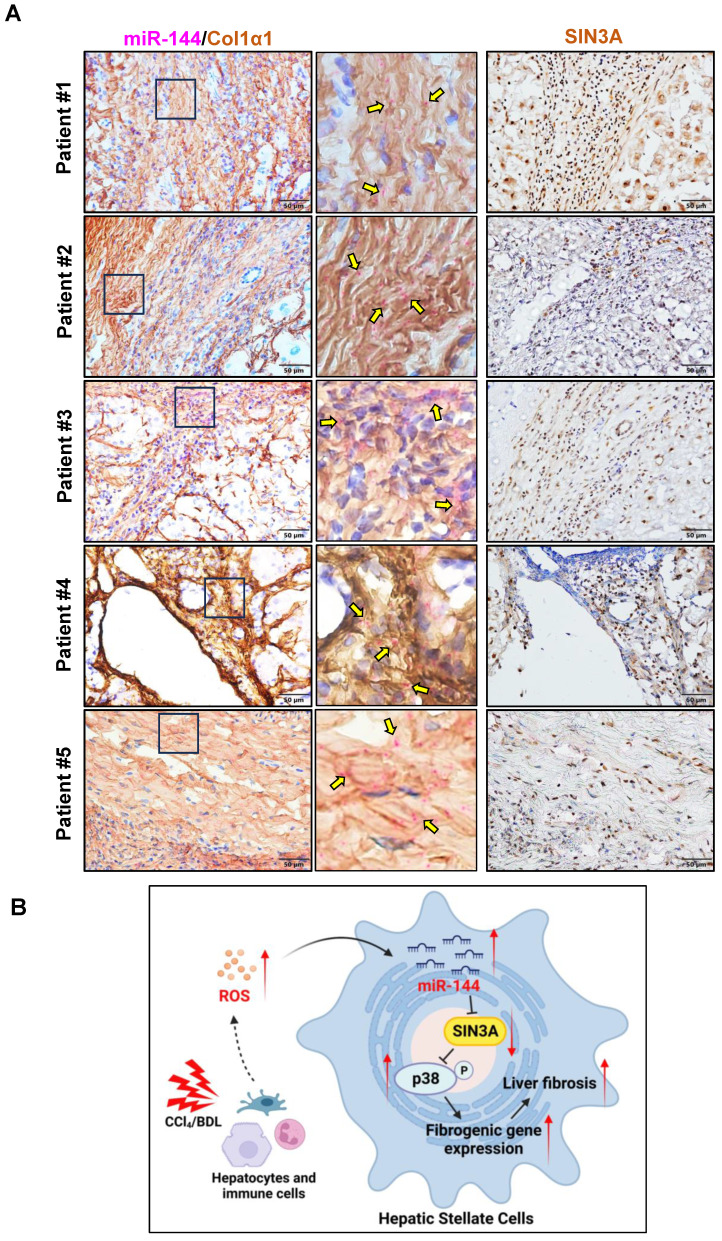
** The significant elevation of miR-144 in HSCs is observed in patients with liver fibrosis.** (A) Serial sections from human fibrotic samples (n=5) were subjected to miR-144 *in situ* hybridization with RNAscope along with immunohistochemistry staining of Col1α1 (Scale bars: 50 μm) or SIN3A staining (Scale bars: 50 μm). Representative images are shown. The magnified view of the details is shown and the yellow arrows indicate miR-144^+^ areas. (B) **A model depicting the critical role of miR-144 in the development of liver fibrosis.** During chronic liver injury, oxidative stress in inflammatory microenvironment exclusively elevated the levels of miR-144 in HSCs, which fuels HSC activation and liver fibrogenesis by controlling SIN3A-p38 axis. Specific inhibition of miR-144 in HSCs may provide a novel therapeutic strategy for the treatment of liver fibrosis.

The above data strongly suggest that preferential elevation of miR-144 in HSCs fuels its activation, finally leading to the development and progression of liver fibrosis. To further investigate whether such elevation of miR-144 is also observed in clinically relevant patients. We performed *in situ* hybridization analyses to detect hepatic miR-144 expression in human samples from patients with liver cirrhosis. As shown in Fig. 10A, as expected, miR-144 was obviously observed in Col1α1^+^ HSCs in human fibrotic samples. Additionally, immunohistochemistry staining from serial sections demonstrated that SIN3A protein levels were also increased in patients with liver fibrosis.

## Discussion

Oxidative stress in liver fibrotic environment is characterized to be one of the crucial initiating factors of liver fibrosis through trigging hepatocyte damage, stimulating the production of profibrogenic mediators from Kupffer cells and circulating inflammatory cells, and directly activating HSCs. Given that HSCs are the primary source of activated ECM-producing cells, unmet need is to deep understand the exact molecular mechanism of initiation and maintenance of HSC activation by ROS induction. In the current study, we demonstrated for the first time that oxidative stress-related miR-144 was preferentially expressed in HSCs during liver fibrosis development, eventually fueling HSC activation and liver fibrogenesis by limiting the SIN3A-p38 axis (Fig. 10B).

Despite the expansive knowledge about the cellular and molecular biology of liver fibrogenesis, this knowledge is mainly HSC-centric. Recently, single-cell RNA sequencing (scRNAseq) analyses provided unprecedented insights into cell-to-cell interaction, emphasizing the importance of liver fibrotic microenvironment in the development and progression of liver fibrosis 33, 34. ROS are short-lived, highly electrophilic molecules, generated in oxygen-related redox reactions during biological process. ROS is one of the typical features in liver fibrotic microenvironment. High cellular levels of ROS cause HSC death; however, non-toxic levels of ROS trigger HSC activation and proliferation. Although oxidative stress seems to have a clear role in HSC activation, the exact mechanism remains obscure. Indeed, miR-144 is remarkedly elevated in HSCs after H_2_O_2_ exposure. Most importantly, this elevation preferentially exists in HSCs of mice with liver fibrosis and human liver fibrotic samples by performing miR-144 *in situ* hybridization with RNAscope probe. This is consistent with previous reports, which found that conditions such as H_2_O_2_ or hypoxia could upregulate the expression of miR-144 14, 15. Since the release of ROS by neutrophils through "oxidative burst" is the dominant source of ROS during liver injury, we isolated neutrophils from mouse bone marrow and tried to perform the co-culture of neutrophils with HSCs. Contrary to our expectations, *in vitro* co-culture experiments found that neutrophils failed to directly drive the enhanced expression of miR-144 in HSCs (data not shown). Neutrophils may suppress miR-144 expression through other unknown mechanisms, as studies have reported that neutrophils were involved in the regression of liver fibrosis 35, 36. However, the underlying mechanism of miR-144 elevation induced by H_2_O_2_ needs to be further investigated in the future.

Another striking finding of the current study is that we identified the functional roles of miR-144 in liver fibrosis *in vivo* and *in vitro*. By far, miR-144 is a well-documented oxidative stress-sensitive miRNA involved in the development of various liver diseases, such as NAFLD and HCC, by inhibiting Nrf2 14, 37. However, the role of miR-144 in HSCs during liver fibrosis has not been illustrated. Our gene manipulation experiments *in vitro* confirmed that overexpression of miR-144 in HSCs could promote its activation. Afterward, we intended to generate HSC-specific miR-144 knockout mice (LRAT-Cre, miR-144^fl/fl^) to investigate its function to verify this notion *in vivo*. Regrettably, due to the proximity of miR-144 and the isogenic cluster miR-451, miR-144^fl/fl^ mice could not be established. Fortunately, emerging evidence revealed that AAV6 exhibited organ tropism for activated HSCs in mice 25, 26; and then we confirmed the targeting efficiency of AAV6 in HSCs by immunofluorescent staining. There are several direct pieces of evidence we provided that miR-144 plays a crucial role in liver fibrosis progression. First, blockade of miR-144 in HSCs *in vivo* significantly attenuates CCl_4_-induced liver fibrosis, whereas overexpression of miR-144 in HSCs results in the adverse phenotype. Second, identical genetic manipulation experiments are also performed in the BDL model, and we found that miR-144 could also expedite the progression of BDL-induced liver fibrosis. All these data support the conclusion that specific inhibition of miR-144 in HSCs may provide a novel therapeutic strategy for the treatment of liver fibrosis.

Next, we have identified that *Sin3a* is the target of miR-144 in HSCs. Our *in vivo* and *in vitro* data suggested that miR-144 significantly downregulated the expression of SIN3A in HSCs. SIN3A is a ubiquitously expressed transcriptional repressor, whose transcriptional regulatory function has been well elucidated in various diseases 38, 39.

Nevertheless, the involvement of SIN3A in liver fibrosis is debated and controversial at present. A previous study found that KLF11 inhibited Smad7 by recruiting *Sin3a* to its promoter, thereby enhancing TGF-β signaling pathway and exacerbating liver fibrosis 40; whereas another study confirmed that NR4A1 limited the degree of liver fibrosis by recruiting Sin3a repressor to the TGF-β target genes 32. Our data demonstrated that suppression of *Sin3a* expression in HSCs significantly accelerated the activation of HSCs *in vitro* and immensely exacerbated the degree of liver fibrosis *in vivo*. Besides, we found that p38 MAPK signaling was tremendously activated after knockdown of *Sin3a*. It is generally acknowledged that p38 is activated by oxidative stress or inflammatory cytokines, and participates in the regulation of biological processes such as cell stress, inflammation, and apoptosis 41, 42. Here, we provide evidence that overexpression of miR-144 or knockdown of *Sin3a* significantly activates p38, leading to the activation of HSCs. In addition, the p38 inhibitor could significantly abolish the induction of HSC activation by miR-144. However, the specific mechanism by which *Sin3a* regulates p38 phosphorylation remains obscure. Notably, previous studies have revealed that knockdown of *Sin3a* resulted in the elevated Rho expression, which was a well-documented upstream molecule involved in the regulation of p38 signaling pathway 43. It is widely accepted that Rho, driven by oxidative stress or pro-inflammatory cytokines, activates MAPKKKs-MKK3/MKK6 cascade, thereby phosphorylating p38 44-46. It is reasonable to speculate that knockdown of *Sin3a* may lead to an augment in Rho expression, thereby activating p38.

In conclusion, under the strike of CCl_4_ or BDL, oxidative stress drives miR-144 to aggravate liver fibrosis by inhibiting the SIN3A-p38 axis. Although the development of miRNA drug candidates is often terminated due to insufficient drug efficacy or lack of safety, the efficacy and safety of miRNA drugs have been greatly improved with the maturity of nanoparticle and exosome delivery technologies 47, 48. Based on the above findings, restrain of miR-144 in HSCs may provide a novel and potential anti-fibrotic strategy.

## Supplementary Material

Supplementary figures and tables.

## Abbreviations

Acta2: actin alpha 2
AAV6: Adeno-Associated Virus 6
BDL: Bile Duct Ligation
CCl_4_: Carbon tetrachloride
Col1α1: Collagen type 1 alpha 1
Col3α1: Collagen type 3 alpha 1
FN1: fibronectin 1
HSC: hepatic stellate cell
H_2_O_2_: hydrogen peroxide
miR-144: microRNA-144
mRNA: messenger RNA
NAFLD: nonalcoholic fatty liver disease
NASH: nonalcoholic steatohepatitis
ROS: Reactive oxygen species
RT-qPCR: reverse transcription quantitative PCR
SIN3A: SIN3 transcription regulator family member A
α-SMA: α-smooth muscle actin
