# Supplementary material for: Oxidative stress promotes liver fibrosis by modulating the microRNA-144 and SIN3A-p38 pathways in hepatic stellate cells
Source: Int J Biol Sci. 2024 Apr 8;20(7):2422–39. doi: 10.7150/ijbs.92749 (PMC11077365; doi:10.7150/ijbs.92749)
Supplement: Supplementary file 1 — Supplementary figures and tables. [file ijbsv20p2422s1.pdf]

Supporting Figure S1

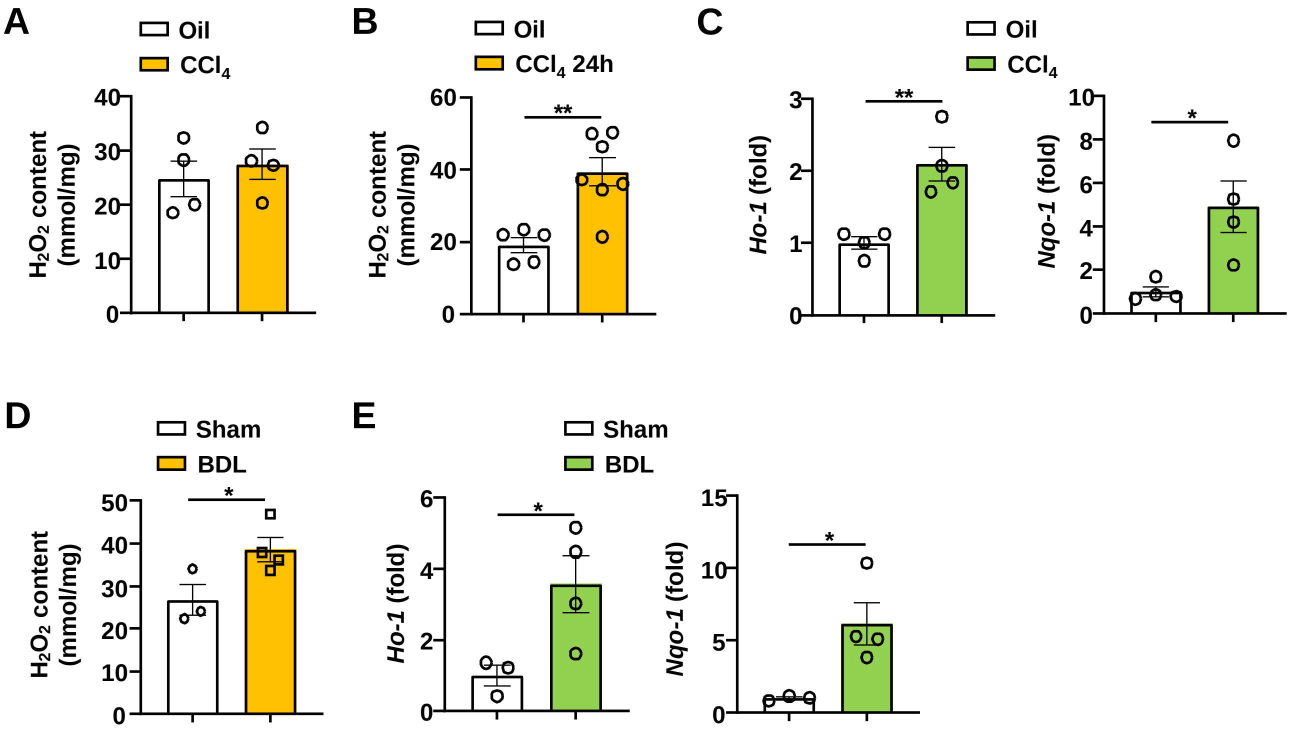

Supporting Figure S1. Hepatic oxidative stress was elevated in the liver of mice with CCl_4 and BDL-induced liver fibrosis. (A, C) C57BL/6J mice were injected intraperitoneally with 2 ml/kg weight of 10% CCl_4 in olive oil twice a week for 6 weeks and were sacrificed 48 hours after the last injection of CCl_4. (A) Hepatic H_2O_2 content was measured. (B) C57BL/6J mice were injected intraperitoneally with 2 ml/kg weight of 10% CCl_4 for 24 hours. Hepatic H_2O_2 content was measured. (C) RT-qPCR analyses of several oxidant related genes. (D-E) C57BL/6J mice were performed BDL or sham operation for 14 days. (D) Hepatic H_2O_2 content was measured. (E) The expression of *Ho-1* and *Nqo-1* were analyzed by RT-qPCR analyses. $^*P < 0.05$, $^{**}P < 0.01$.

Supporting Figure S2

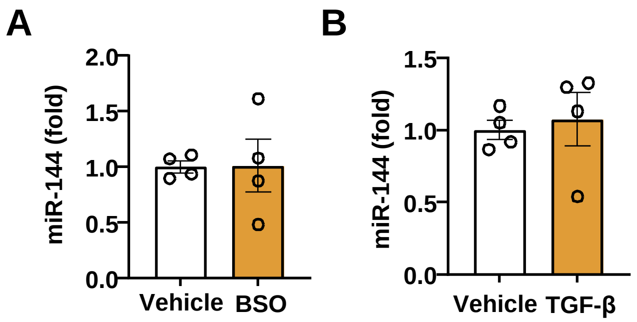

Supporting Figure S2. BSO and TGF-β did not elevated miR-144 in HSCs. (A) LX-2 cell lines were administered with BSO (500μM). The expression of miR-144 were analyzed by RT-qPCR analyses. (B) LX-2 cell lines were administered with TGF-β (10ng/ml). The expression of miR-144 were analyzed by RT-qPCR analyses.

Supporting Figure S3

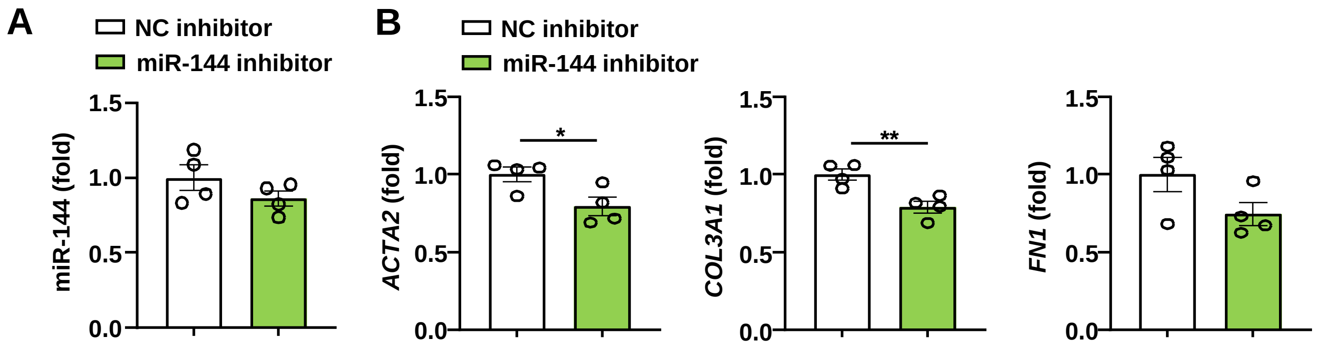

Supporting Figure S3. Blockage of miR-144 in LX-2 cells inhibits HSC activation. LX-2 cell lines were transfected with miR-144 inhibitor and NC inhibitor (100nM) for 48 hours. (A) The expression of miR-144 were analyzed by RT-qPCR analyses. (B) The expression of *ACTA2*, *COL3A1* and *FN1* were analyzed by RT-qPCR analyses. *$P < 0.05$, **$P < 0.01$.

Supporting Figure S4

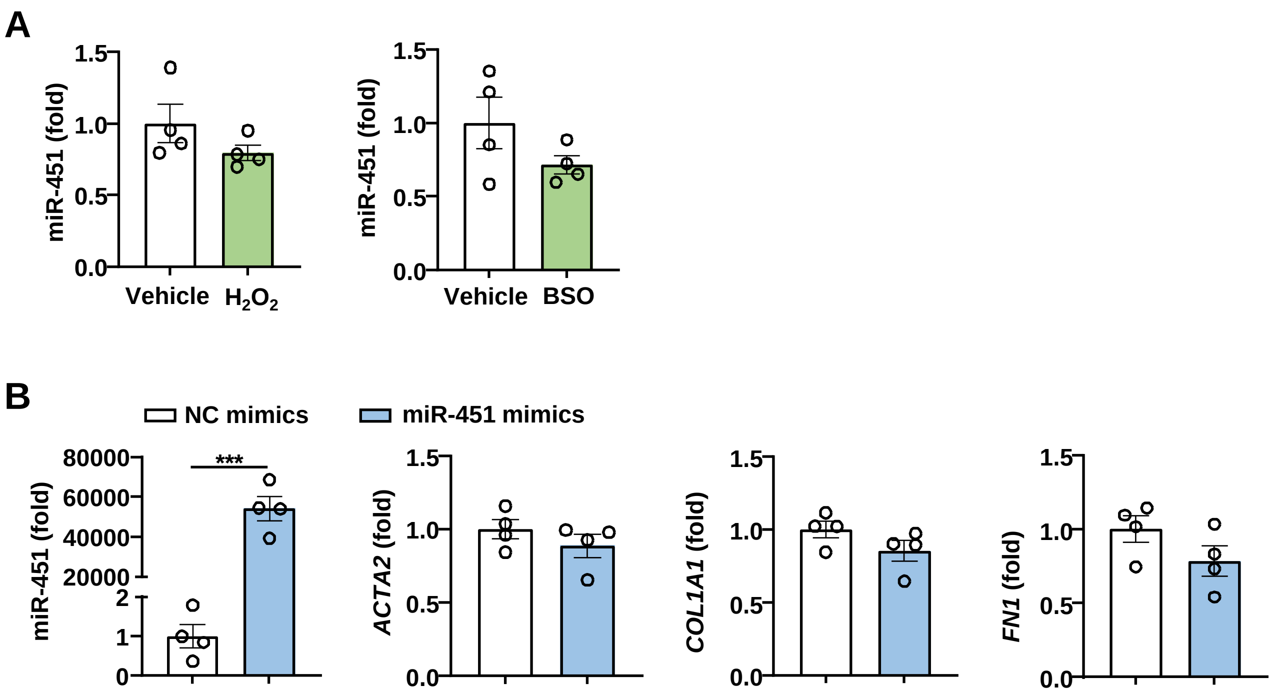

Supporting Figure S4. miR-451 is not involved in HSC activation. (A) LX-2 cell lines were administered with BSO (500μM) or H_2O_2 (200μM) for 3 hours. The expression of miR-451 were analyzed by RT-qPCR analyses. (B) LX-2 cell lines were transfected with miR-451 mimics and NC mimics (20nM) for 48 hours. The expression of miR-451, *ACTA2*, *COL1A1* and *FN1* were analyzed by RT-qPCR analyses. ***$P < 0.001$.

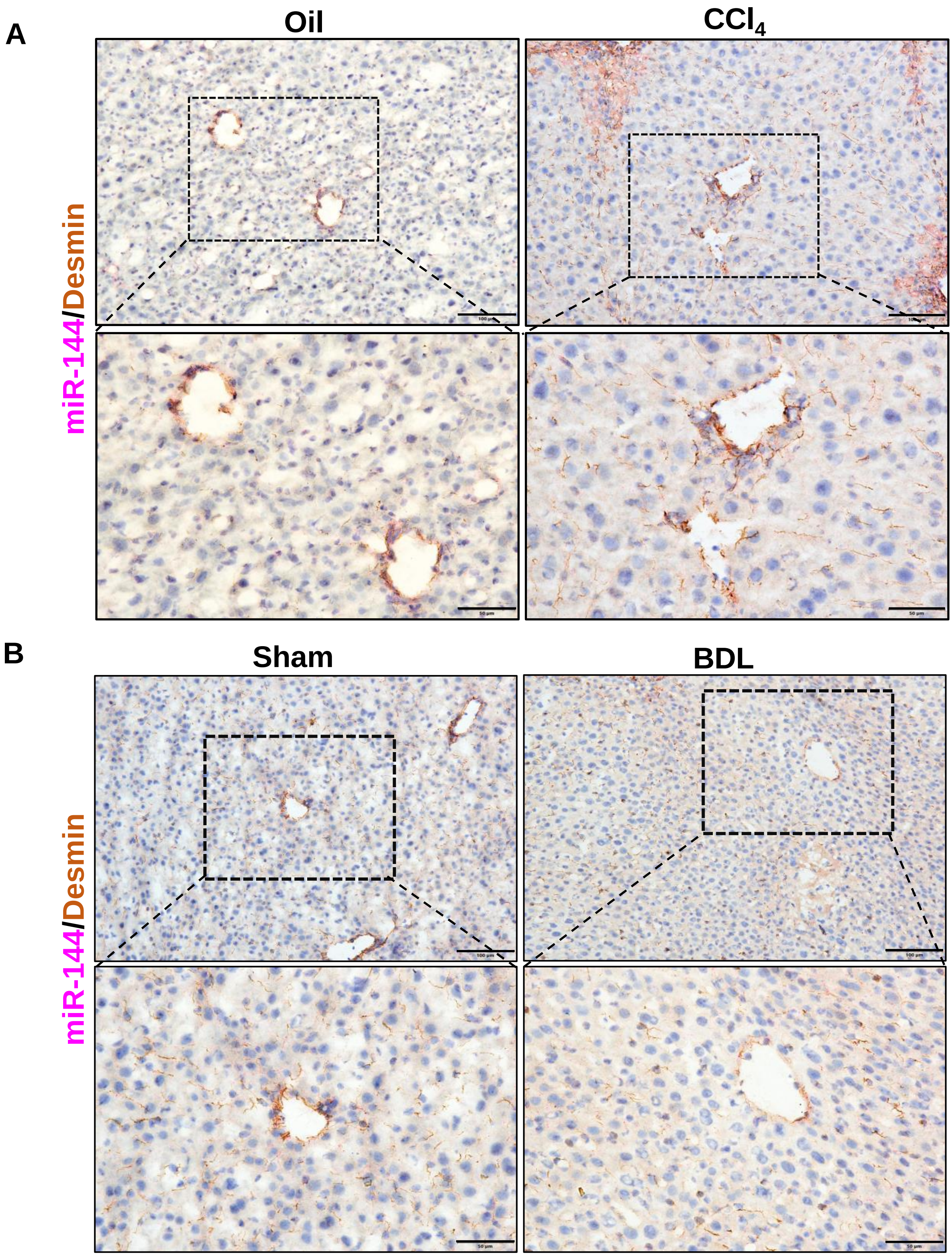

Supporting Figure S5. miR-144 is not elevated in non-fibrotic regions in liver fibrosis mouse models. C57BL/6J mice were injected intraperitoneally with 2 ml/kg weight of 10% CCl_4 in olive oil twice a week for 6 weeks or performed BDL operation for 2 weeks. Liver tissue samples were collected. Frozen liver tissue sections were subjected to miR-144 in situ hybridization with RNAscope probe. (A) Representative images of miR-144 expression in non-fibrotic regions in CCl_4 mouse model are shown. Scale bars: 100 μm or 50 μm. (B) Representative images of miR-144 expression in non-fibrotic regions in BDL model are shown. Scale bars: 100 μm or 50 μm.

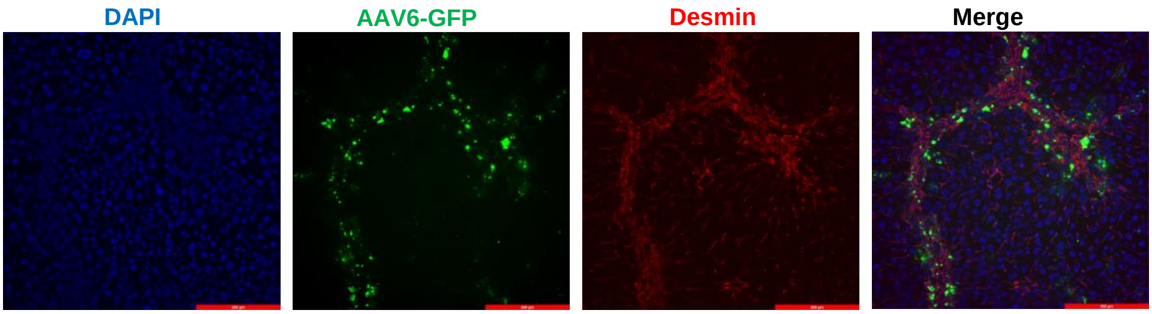

Supporting Figure S6. AAV6 mainly infects HSCs. C57BL/6J mice were injected intraperitoneally with 2 ml/kg weight of 10% CCl_4 in olive oil twice a week for 2 weeks before administering with AAV6-GFP. After AAV6 injection, another 8 doses of CCl_4 were administered to the mice, which were sacrificed 48 hours after the last injection of CCl_4. (A) Representative images of GFP (green), Desmin (red), and nuclei (blue) are shown. Scale bars: 200 μm.

Supporting Figure S7

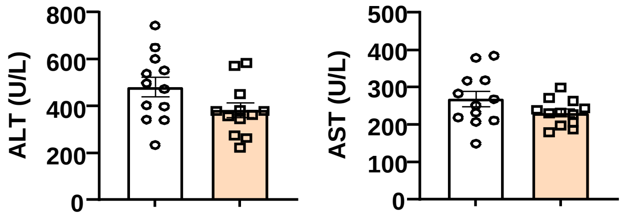

Supporting Figure S7. Blockade of miR-144 in hepatic stellate cells did not affect liver injury. C57BL/6J mice were administrated with AAV6-miR-144 antagomir. Serum ALT and AST levels were measured.

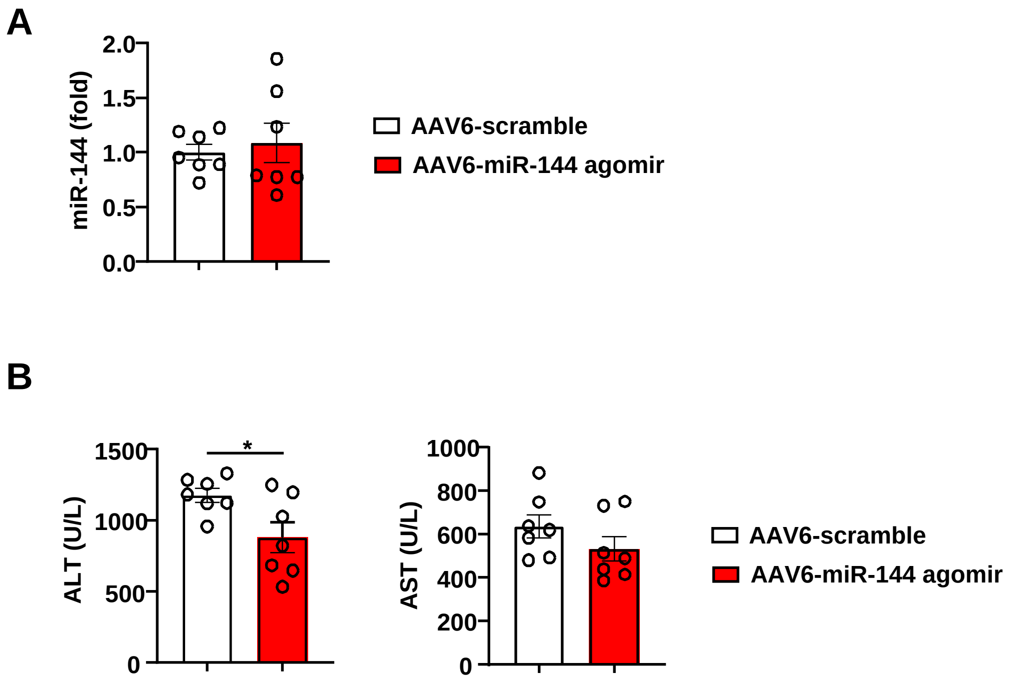

Supporting Figure S8. The liver injury after overexpression of miR-144 in HSCs. (A) The expression of miR-144 in liver were analyzed by RT-qPCR analyses. (B) Serum ALT and AST levels were measured. *$P < 0.05$.

Supporting Figure S9

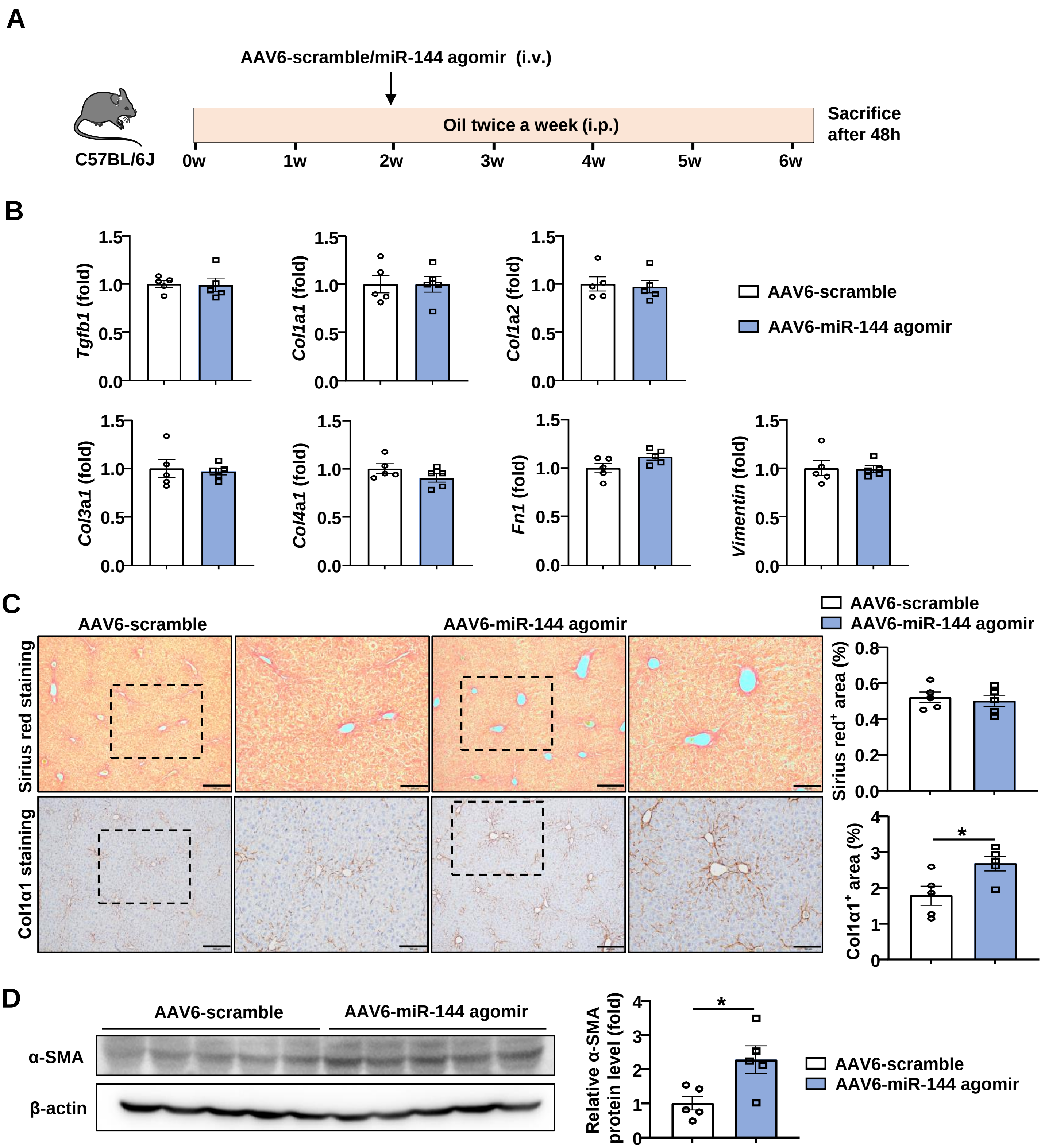

Supporting Figure S9. Overexpression of miR-144 in HSCs barely influences the development of liver fibrosis. (A) Schematic overview of the experimental design. Liver tissue samples were collected. (B) RT-qPCR analyses of several hepatic fibrogenesis genes. (C) Liver tissues were subjected to Sirius red staining (Scale bars: 200 μm or 100 μm) and Col1α1 staining (Scale bars: 200 μm or 100 μm). (D) α-SMA expression was analyzed by Western blot. Values represent means ± SEM (n=5 per group).

Supporting Figure S10

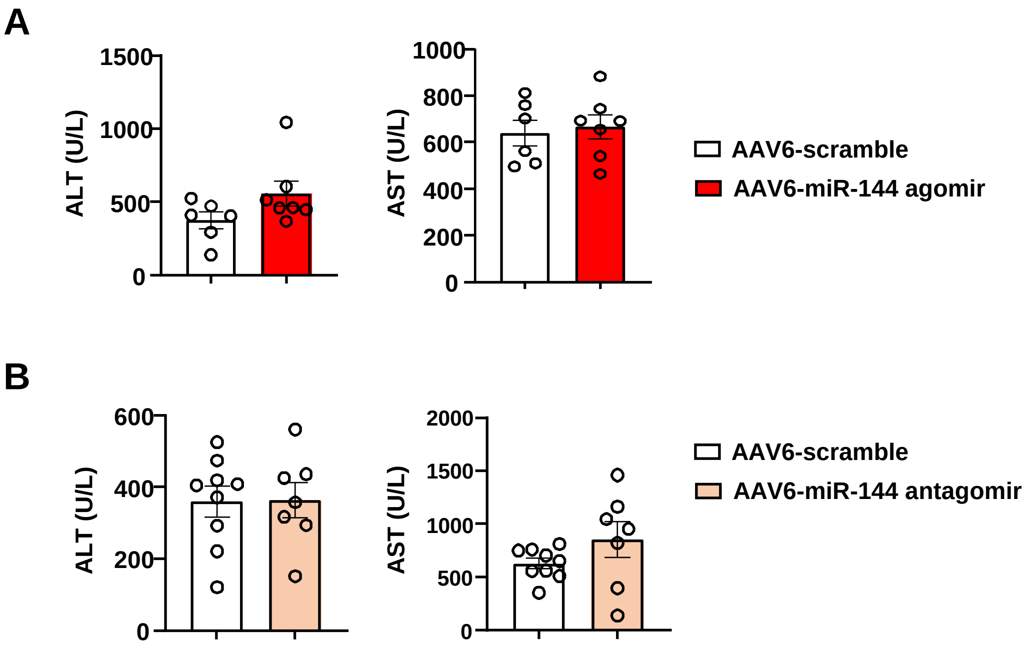

Supporting Figure S10. Overexpression or inhibition of miR-144 in hepatic stellate cells did not affect liver injury. (A-B) C57BL/6J mice were administrated with AAV6-miR-144 agomir/antagomir after bile duct ligation. Then the mice were sacrificed 17 days later and their serum was collected. Serum ALT and AST levels were measured.

Supporting Table S1. The list general characteristics of the liver fibrosis human samples.

Patient #	Gender	Age	Primary Diagnosis	ALT (IU/L)	AST (IU/L)
1	F	63	Liver fibrosis, autoimmune hepatitis	60	73
2	F	58	Cirrhosis, CHB, HCC	109	371
3	M	49	Cirrhosis, CHB, HCC	28	61
4	F	30	Liver fibrosis, autoimmune hepatitis	11	19
5	M	61	Cirrhosis, HCC	33	119

CHB: chronic hepatitis B; HCC: hepatocellular carcinoma

Supporting table S2: RT-qPCR primer sequences

**Genes (mouse)**	**Forward primer (5'-3')**	**Reverse primer (5'-3')**
18s	AACTTTCGATGGTAGTCGCCGT	TCCTTGGATGTGGTAGCCGTTT
Acta2	TCCTGACGCTGAAGTATCCGATA	GGTGCCAGATCTTTTCCATGTC
Col1a1	TAGGCCATTGTGTATGCAGC	ACATGTTCAGCTTTGTGGACC
Col1a2	GGTGAGCCTGGTCAAACGG	ACTGTGTCCTTTCACGCCTTT
Col3a1	TAGGACTGACCAAGGTGGCT	GGAACCTGGTTTCTTCTCACC
Col4a1	CACATTTTCCACAGCCAGAG	GTCTGGCTTCTGCTGCTCTT
Fn1	TTCAAGTGTGATCCCCATGAAG	CAGGTCTACGGCAGTTGTCA
Vimentin	TCCACACGCACCTACAGTCT	CCGAGGACCGGGTCACATA
Tgfb1	CAACCCAGGTCCTTCCTAAA	GGAGAGCCCTGGATACCAAC
Sin3a	AGCCGAGTGTCCCAGCTATT	TCTGCACCTCAATTTTGTAGCC
Dusp1	GTTGTTGGATTGTCGCTCCTT	TTGGGCACGATATGCTCCAG
Hgf	ACTTCTGCCGGTCCTGTTG	CCCCTGTTCCTGATACACCT
Gsk3b	TGGCAGCAAGGTAACCACAG	CGGTTCTTAAATCGCTTGTCCTG
Foxo1	GGGTCCCACAGCAACGATG	CACCAGGGAATGCACGTCC
Cav2	TCACCAGCTCAACTCTCATCT	GCCAGAAATACGGTCAGGAACT
Pten	TGGATTCGACTTAGACTTGACCT	GCGGTGTCATAATGTCTCTCAG
Tgif2	ATGTCGGACAGCGATCTAGG	TCCCGGAGGATCTTTACTGAC
Ho-1	AGGTACACATCCAAGCCGAGA	CATCACCAGCTTAAAGCCTTCT
Nqo-1	AGGATGGGAGGTACTCGAATC	AGGCGTCCTTCCTTATATGCTA

**Genes (human)**	**Forward primer (5'-3')**	**Reverse primer (5'-3')**
18s	GGCCCTGTAATTGGAATGAGTC	CCAAGATCCAACTACGAGCTT
ACTA2	GTGACGAAGCACAGAGCAAA	CTTTTCCATGTCGTCCCAGT
COL1A1	CAGATCACGTCATCGCACAA	TGTGAGGCCACGCATGAG
COL3A1	AGGACTGACCAAGATGGGAA	AGGGGAGCTGGCTACTTCTC
COL4A1	CCTTTTGTCCCTTCACTCCA	CTCCACGAGGAGCACAGC
FN1	CGGTGGCTGTCAGTCAAAG	AAACCTCGGCTTCCTCCATAA
TGFB1	CAATTCCTGGCGATACCTCAG	GCACAACTCCGGTGACATCAA
SIN3A	GGTGGAGGATGCGCTATCTTA	GGGTGTCGATGCTCTGAGATTT

Supporting table S3: miRNA primer sequences

miRNA	RT primer	qPCR primer
Sno202	GTCGTATCCAGTGCAGGGTCCGAGGTATTCGCACTGGATACGACCATCAG	AAGTACTTTTGAACCCTTTTCCAT
RUN44	GTCGTATCCAGTGCAGGGTCCGAGGTATTCGCACTGGATACGACAGTCAG	GAACATGAAGGTCTTAATTAGCTCTAA
miR-144	GTCGTATCCAGTGCAGGGTCCGAGGTATTCGCACTGGATACGACAGTACA	GCGCGCGTACAGTATAGATGA
miR-451a	GTCGTATCCAGTGCAGGGTCCGAGGTATTCGCACTGGATACGACAACTCA	CGCGAAACCGTTACCATTAC
